# Light Induced Cobalt(III) Carbene Radical Formation from Dimethyl Malonate As Carbene Precursor

**DOI:** 10.1021/acs.organomet.4c00127

**Published:** 2024-05-24

**Authors:** Demi D. Snabilié, Rens Ham, Joost N. H. Reek, Bas de Bruin

**Affiliations:** Van ‘t Hoff Institute for Molecular Sciences, University of Amsterdam, Science Park 904, Amsterdam 1098 XH, The Netherlands

## Abstract

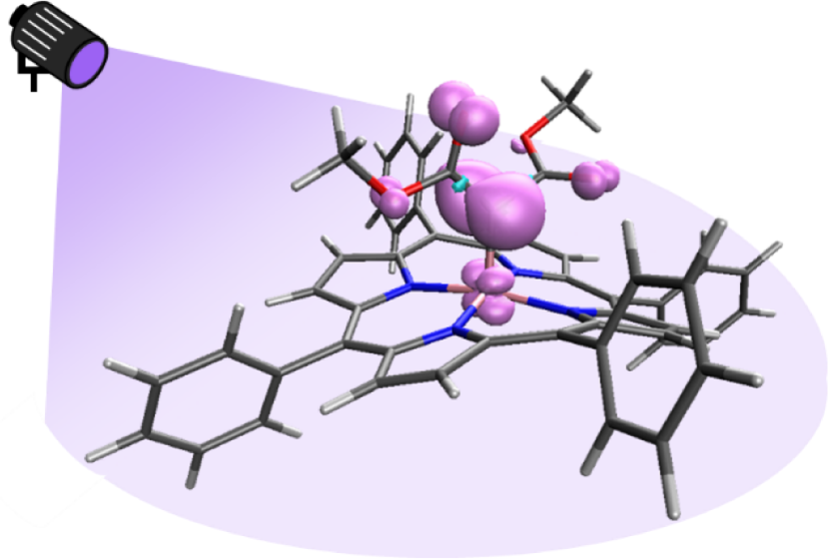

Radical-type carbene transfer catalysis is an efficient method for the direct functionalization of C–H and C=C bonds. However, carbene radical complexes are currently formed via high-energy carbene precursors, such as diazo compounds or iodonium ylides. Many of these carbene precursors require additional synthetic steps, have an explosive nature, or generate halogenated waste. Consequently, the utilization of carbene radical catalysis is limited by specific carbene precursors that access the carbene radical intermediate. In this study, we generate a cobalt(III) carbene radical complex from dimethyl malonate, which is commercially available and bench-stable. EPR and NMR spectroscopy were used to identify the intermediates and showed that the cobalt(III) carbene radical complex is formed upon light irradiation. In the presence of styrene, carbene transfer occurred, forming cyclopropane as the product. With this photochemical method, we demonstrate that dimethyl malonate can be used as an alternative carbene precursor in the formation of a cobalt(III) carbene radical complex.

## Introduction

Carbene transfer catalysis is an efficient tool for the production of fine chemicals as it enables the direct synthesis of C–C bonds. Many methodologies have been developed that involve carbene transfer catalysis,^1^ such as olefin metathesis,^2^ alkene polymerization,^3^ and C–H or X–H insertion reactions.^1d,4,5^ While most carbene transfer reactions proceed via a Fischer- or Schrock-type carbene intermediate,^6,7^ the use of radical-type carbene transfer catalysis has emerged over the past decades.^8^ These reactions involve a metal carbene radical complex, which is often cobalt,^9^ but other metals have been reported as well.^10^ A metal carbene radical complex can be best described as a Fischer carbene that is one-electron reduced.^11^ This one-electron reactivity enables direct functionalization of C–H bonds and olefins in various reactions, such as the formation of 5-, 6-, and 8-membered rings,^12−14^ and cyclopropanation reactions, which have been most reported in literature.^15^

Zhang and coworkers reported multiple asymmetric cyclopropanation reactions with diazo compounds as the carbene precursor.^16^ The reaction proceeded via a cobalt(III) carbene radical complex that was formed after the elimination of N_2_ from the diazo compound. Using an asymmetric cobalt porphyrin complex, they were able to perform these radical-type cyclopropanation reactions with excellent enantioselectivity. The respective diazo compound can also be formed *in situ* from a hydrazone under basic conditions to avoid high concentrations.^9a,17^ Next to diazo compounds, iodonium ylides have been reported as efficient carbene precursors for the formation of carbene radical complexes,^18^ which has been studied in our group in the cyclopropanation reaction with styrene.^19^ It was found that with an excess of iodonium ylide, a biscarbene complex was formed that transferred the carbene moiety onto the double bond, forming cyclopropane with high yields and short reaction times (up to 99%, < 5 min). In this work, the general sensitivity of metal carbene radical complexes toward deactivation was also observed: most metal carbene radical complexes are very competent in abstracting a hydrogen from weak C–H bonds.^20^ Contrarily, the group of Che showed that the hydrogen atom transfer (HAT) reaction can actually be used to generate the desired active radical species, thereby leading to product formation rather than deactivation.^21^

**Figure 1:**
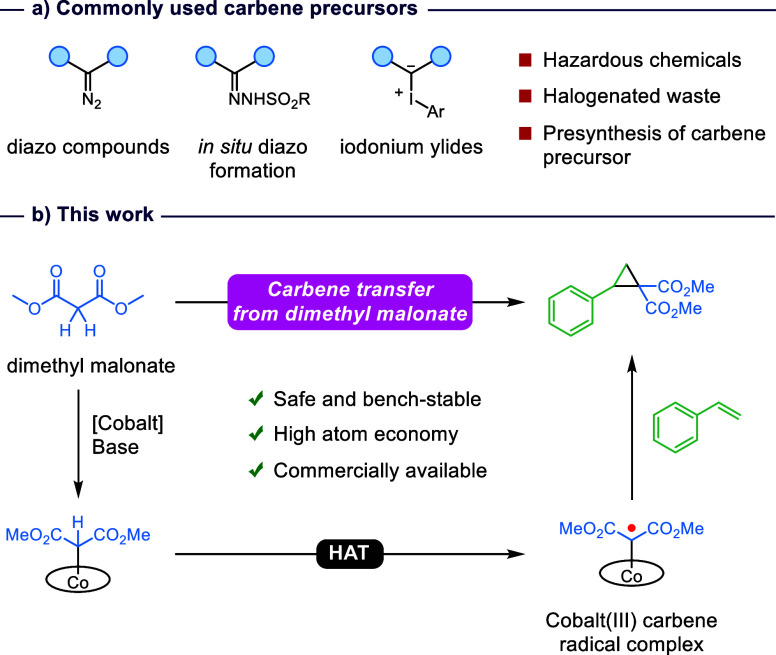
a) Commonly used carbene precursors in (radical-type) carbene transfer catalysis. b) Proposed radical-type carbene transfer from dimethyl malonate via a cobalt(III) carbene radical complex upon light irradiation.

However, all of the above-mentioned reactions require high-energy carbene precursors bearing excellent leaving groups to generate the reactive carbene radical intermediate (Figure 1a). These carbene precursors have to be separately synthesized, generate halogenated waste, and have toxic and explosive properties. As a consequence, the requirement of these specific carbene precursors limits the utilization of radical-type carbene transfer catalysis and thus the direct functionalization of carbon bonds. Hence, our research focuses on the use of an alternative carbene precursor that is bench-stable and readily available but enables the same one-electron reactivity. Active methylene compounds (that is, bearing electron withdrawing groups) have been used in various cyclopropanation reactions,^22^ replacing the hazardous but commonly used diazo compounds. The group of Xu reported the intramolecular electrochemical cyclopropanation reaction with alkene-tethered α-cyanoamides.^22c^ They were able to produce a wide variety of products, including lactams, lactones, and cyclic ketones. More recently, the photocatalytic cyclopropanation reaction of olefins was reported by Giri and coworkers.^23^ Using active methylene compounds as starting reagents and under aerobic photochemical conditions, the cyclopropanation reaction could be performed in good yield with various substrates. Only a catalytic amount of iodine was used to reduce the photocatalyst (4CzIPN) back to its ground state, thereby closing the catalytic cycle. This photochemical method could even be used to form various pharmaceuticals and natural products in a single step. Despite the successful formation of cyclopropanes from active methylene compounds, the formation of metal carbene radical complexes via this starting material has not yet been reported. This is of relevance to further developing (enantioselective) carbene transfer reactions from active methylene compounds in the future.

Inspired by the above-mentioned studies, we hypothesized that dimethyl malonate, an active methylene compound, can be used as a carbene precursor for the generation of a cobalt(III) carbene radical complex (Figure 1b). Under basic conditions, dimethyl malonate will be deprotonated, and the respective anion can then coordinate the cobalt complex. Normally, this species is a deactivated product resulting from HAT by the carbene radical complex, but with the work of Che in mind, we postulated that HAT could lead to the desired cobalt(III) carbene radical complex. Benzophenone is known as an excellent HAT reagent and was, therefore, selected for this study.^24^ The formed cobalt(III) carbene radical complex can react directly with an olefin to form a cyclopropane as the product. In this work, we demonstrate that dimethyl malonate can be used as a carbene precursor to generate a cobalt(III) carbene radical complex, which is characterized by EPR and NMR spectroscopy.

## Results and Discussion

We started performing the cyclopropanation reaction between dimethyl malonate and styrene to investigate whether dimethyl malonate can be used as a carbene precursor. Previous studies showed that cobalt porphyrin complexes have excellent reactivity for this reaction.^15,19^ Hence, we selected [Co^II^(TPP)] and [Co^III^(TPP)Cl] as potential catalysts. Furthermore, KO*t*Bu was used as a non-nucleophilic base to deprotonate dimethyl malonate and benzophenone as a HAT reagent, which was activated with a 370 nm light source. After irradiating the reaction mixture with [Co^III^(TPP)Cl] (10 mol%) overnight, cyclopropane **3** was observed with an average yield of 11% (Table 1, entry 1), which is a stoichiometric (∼100%) conversion with respect to the cobalt catalyst. However, when the reaction was performed with [Co^II^(TPP)], no product was formed (entry 2), showing that the oxidation state of cobalt plays a crucial role in the reaction. Control experiments showed no product formation in the absence of [Co^III^(TPP)Cl], KO*t*Bu, and light (entry 3–5). Only in absence of benzophenone, traces of product were observed (entry 6), suggesting that the product can also be formed without benzophenone as HAT reagent. These results confirm our hypothesis that dimethyl malonate can be used as carbene precursor in the cyclopropanation reaction. If the reaction proceeds via a cobalt(III) carbene radical intermediate that reacts with styrene, then cobalt(III) is known to be reduced back to cobalt(II).^19^ In contrast to [Co^III^(TPP)Cl], [Co^II^(TPP)] is unlikely to bind and activate dimethyl malonate due to its lower oxidation state. Thus, when the cobalt catalyst is not regenerated back to its formal 3+ oxidation state after the reaction, cyclopropane **3** is formed only stoichiometrically (entry 1). Attempts to oxidize cobalt(II) back to cobalt(III) under the applied reaction conditions in the presence of all substrates were unfortunately unsuccessful (see Table S2).

**Table 1:**
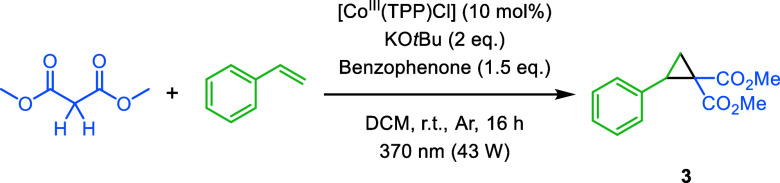
Initial and Control Experiments in the Carbene Transfer Cyclopropanation Reaction from Dimethyl Malonate^a^

entry	deviations	yield
**1**	none	11% ± 2%^b^
**2**	[Co^II^(TPP)]	0%
**3**	no [Co^III^(TPP)Cl]	0%
**4**	no KO*t*Bu	0%
**5**	dark	0%
**6**	no benzophenone	<5%

a Conditions: dimethyl malonate (0.05 mmol, 1 eq.), [Co] (10 mol%), styrene (5 eq.), benzophenone (1.5 eq.), and KO*t*Bu (2 eq.) were mixed in dry DCM (1 mL) under argon and stirred overnight at r.t. with light irradiation of 370 nm (43 W). Yields are based on ^1^H NMR analysis of the crude products with 1,3,5-tritertbutylbenzene or 1,3,5-trimethoxybenzene as an external standard.

b Yield is an average of 6 experiments.

**Scheme 1:**
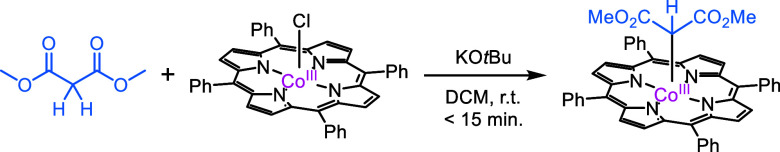
Synthesis of Complex 1, [Co^III^(TPP)(CH(CO_2_Me)_2_], from [Co^III^(TPP)Cl] and Dimethyl Malonate under Basic Conditions.

We were intrigued by the formation of cyclopropane **3** from dimethyl malonate and investigated the mechanism of this reaction further. We postulated that the deprotonated dimethyl malonate reacts with [Co^III^(TPP)Cl] first (Scheme 1), before HAT by benzophenone occurs. Under basic conditions, dimethyl malonate and [Co^III^(TPP)Cl] were mixed, and the formed complex was characterized by NMR spectroscopy and high resolution mass spectrometry (HRMS) (see Supporting Information). The ^1^H NMR spectrum showed a signal at −3.16 ppm, which is indicative for a single proton on a carbon atom that is attached to a cobalt porphyrin complex.^19^ In combination with HRMS measurements that showed a mass of 802.1984 [M+], the formed complex was identified as [Co^III^(TPP)(CH(CO_2_Me)_2_] (complex **1**). Contrarily, by exposing [Co^II^(TPP)] to the same conditions, no adduct was formed as indicated by the lack of signal in the negative region of the ^1^H NMR spectrum (see Figure S9). This confirms that [Co^II^(TPP)] is indeed unable to bind the dimethyl malonate anion.

**Figure 2:**
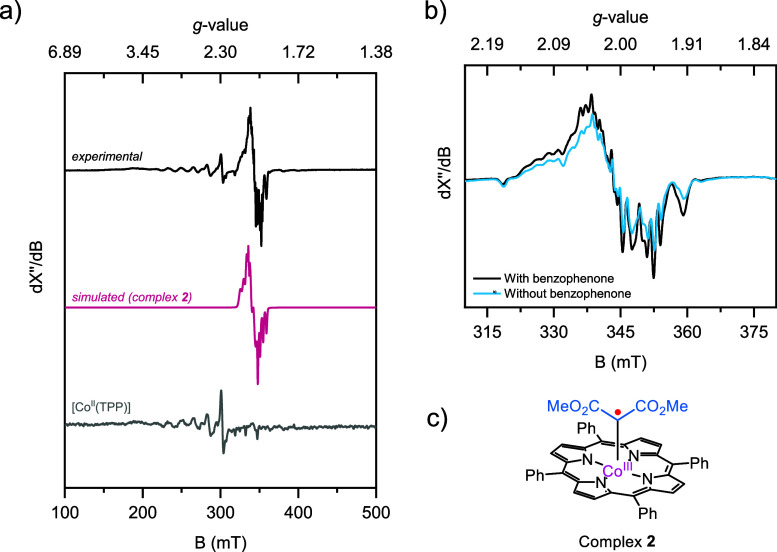
a) X-band EPR spectra obtained after irradiation (370 nm) of *in situ* formed complex **1** and benzophenone (black) and [Co^II^(TPP)] (gray) in toluene-*d*_*8*_. Simulated profile of the cobalt(III) carbene radical complex **2** (pink). b) X-band EPR spectra obtained after irradiation (370 nm) of *in situ* formed complex **1** with benzophenone (black) and without benzophenone (blue) (see Supporting Information for experimental and simulation parameters). c) Schematic structure of cobalt(III) carbene radical complex **2**.

We hypothesized that a cobalt(III) carbene radical complex might be formed after HAT from complex **1**. To observe this carbene radical complex, we used continuous wave X-band EPR spectroscopy at 40 K (Figure 2). Complex **1** was formed *in situ* by mixing [Co^III^(TPP)Cl], dimethyl malonate, and KO*t*Bu in toluene-*d*_*8*_. The solution was filtered and added to benzophenone inside a quartz tube. Next, the EPR sample was irradiated with 370 nm (43 W) at room temperature before freezing the sample immediately. Figure 2a shows the EPR signal obtained after irradiating the sample with light (black line). The signal can be assigned to two species: first, it contains a carbon centered radical that is attached to a cobalt porphyrin, of which the simulated spectrum is depicted below (pink line). The signal corresponds to the previously reported cobalt(III) carbene radical complex (**2**) (Figure 2c).^19^ Second, the obtained experimental spectrum also contains features of [Co^II^(TPP)] (gray line). Under dark conditions, no signal was observed: neither complex **2** nor [Co^II^(TPP)] (see Figure S18). These results indicate that [Co^II^(TPP)] is formed from complex **1** upon irradiation of light. To further investigate the reaction, benzophenone was excluded from the reaction mixture as a control experiment. Again, the *in situ* formed complex **1** was irradiated with light before measuring the EPR spectrum. Figure 2b shows the EPR spectra with (black) and without (blue) benzophenone in the reaction mixture. The same signal, although less intense, was observed when benzophenone was not present in the sample. This demonstrates that cobalt(III) carbene radical complex **2** is also formed without a HAT reagent, only in lower concentrations.

**Figure 3:**
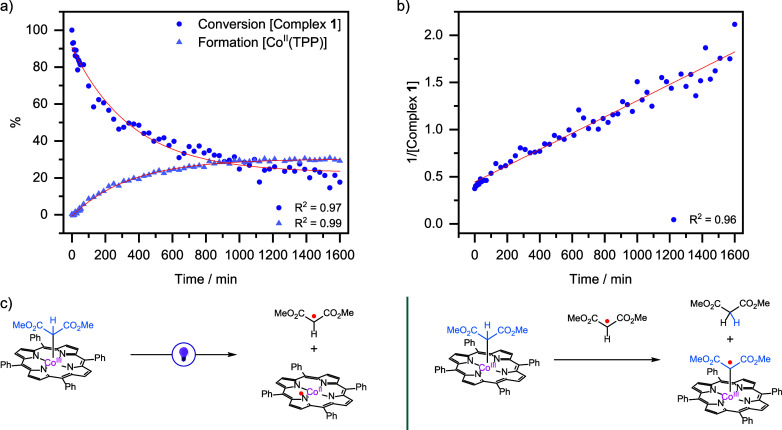
a) Conversion of complex **1** and formation of [Co^II^(TPP)] over time upon light irradiation (390–500 nm) during ^1^H NMR measurements (2.68 mM, in DCM-*d*_*2*_). b) 1/[Complex **1**] plotted against time shows a linear relationship in the conversion of complex **1**. c) Homolytic cleavage of Co–C bond in complex **1**, resulting into [Co^II^(TPP)] and malonate radical (left), of which the latter can perform HAT from complex **1,** leading to the cobalt(III) carbene radical complex **2** and dimethyl malonate (right).

To gain more insight into the formation of the cobalt(III) carbene radical complex **2** under the influence of light, additional experimental and computational studies were performed. In absence of benzophenone, complex **1** was dissolved in DCM-*d*_*2*_, and its conversion was monitored by NMR spectroscopy (Figure 3). Under dark conditions, the concentration of complex **1** remains constant (see Figure S21). Next, complex **1** was irradiated by using a light probe attached to the NMR machine, while the NMR spectra were measured over time. Because complex **1** mainly absorbs within the visible light region (for the UV–vis absorption spectrum, see Figure S8), a 390–500 nm light source was used. We observed that complex **1** decreases exponentially upon the irradiation of light (Figure 3a), indicating that this process is not solely limited by the input of photons. Additionally, the formation of [Co^II^(TPP)] was observed, suggesting that the Co–C bond in complex **1** is homolytically cleaved instead of the C–H bond. This results in the formation of a dimethyl malonate radical and [Co^II^(TPP)] (Figure 3c, left). We suspected that this malonate radical can abstract a hydrogen atom from a second molecule of complex **1** (Figure 3c, right), forming the cobalt(III) carbene radical complex that was observed by EPR spectroscopy (see above). Further evidence for this hypothesis was found when we plotted 1/[Complex **1**] versus time and observed a linear trend in the conversion of complex **1**, which is close to a second order relationship (Figure 3b, R^2^ = 0.96). This suggests that the conversion of complex **1** is influenced by two reactions: (i) the homolytic cleavage of the Co–C bond and (ii) the HAT via the formed malonate radical. However, the first reaction must occur before the second reaction can transpire. Therefore, the derivation of the reaction order kinetics is not unambiguous. Yet, the observed linear relationship indicates that two molecules of complex **1** lead to its conversion.

Thus far, we postulated that next to benzophenone a dimethyl malonate radical can also perform HAT from complex **1**, forming cobalt(III) carbene radical complex **2**. To further study this hypothesis, we turned to density functional theory (DFT) calculations to estimate bond dissociation energies (BDEs) (Table 2). The BDEs indicate whether it is energetically feasible to homolytically cleave the respective bond. The BDE of the Co–C bond in complex **1** is 33 kcal/mol, which is relatively low compared to 95 kcal/mol for the C–H bond in complex **1** (entries 1 and 2, respectively). Hence, the Co–C bond is most likely homolytically cleaved upon irradiation of visible light (390–500 nm ∼ E = 73–57 kcal/mol), forming a dimethyl malonate radical and [Co^II^(TPP)], of which the latter is observed by NMR spectroscopy (see above). Dimethyl malonate has a BDE of 97 kcal/mol (entry 3), meaning that in its radical form, it is strong enough to abstract the hydrogen from the C–H bond in complex **1**, resulting in the cobalt(III) carbene radical complex **2**. According to these DFT calculations, the reactions in Figure 3c are energetically feasible.

**Table 2:**
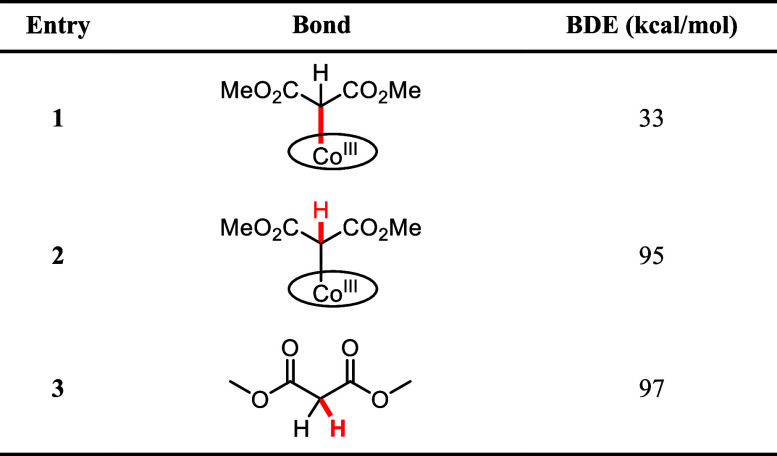
DFT-Calculated Bond Dissociation Energies^a^^b^

a Calculated bonds are highlighted in red.

b Computational details: BP86/def2-TZVP/disp3/m4-grid.

**Scheme 2:**
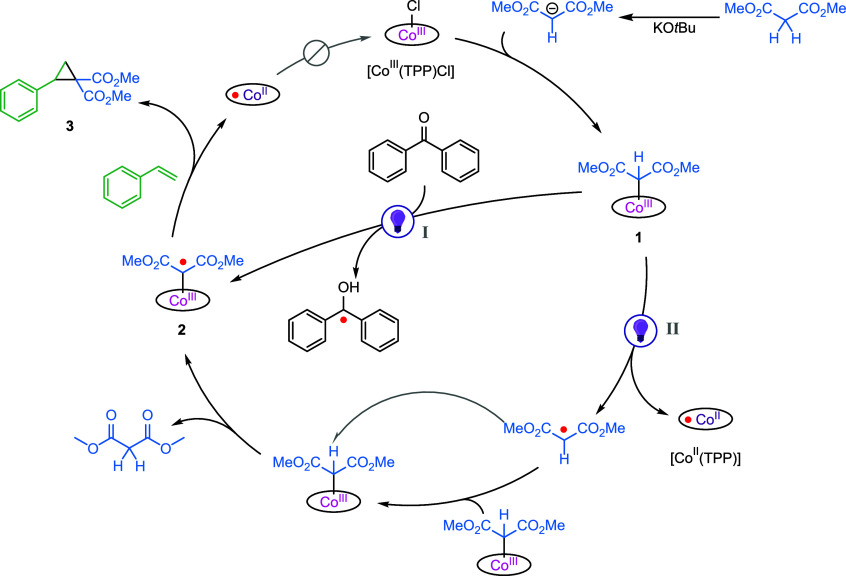
Proposed mechanism for the photochemical cyclopropanation reaction between dimethyl malonate and styrene via a cobalt(III) carbene radical complex.

Based on the above-mentioned results, the mechanism in Scheme 2 is proposed. After deprotonation of dimethyl malonate by KO*t*Bu, the respective anion replaces the chloride atom, forming complex **1**. From here, two pathways can occur: I) benzophenone can abstract the hydrogen atom, forming the desired cobalt(III) carbene radical complex **2**. II) Alternatively, the Co–C bond is homolytically cleaved, resulting in [Co^II^(TPP)] and a dimethyl malonate radical, of which the latter can abstract a hydrogen atom from another molecule of complex **1**. In the presence of styrene, complex **2** reacts with the double bond, forming cyclopropane **3** as the product and [Co^II^(TPP)], which cannot interact with the anionic malonate and, therefore, ends the cycle. In the absence of benzophenone, only a maximum yield of 5% can theoretically be achieved via pathway II, since two molecules of complex **1** are required to generate one molecule of radical complex **2**. Hence, we believe that pathway I is dominant because an average yield of 11% of cyclopropane **3** is observed when using 10 mol% [Co^III^(TPP)Cl].

## Conclusion

In this work, we investigated whether dimethyl malonate could be used as a carbene precursor in the cyclopropanation reaction with styrene. Under photochemical conditions, a cobalt(III) carbene radical complex was formed from dimethyl malonate, and the formation of the desired cyclopropane was observed. The reaction involves a deprotonation by KO*t*Bu followed by HAT, which can be either performed by benzophenone (an additional HAT reagent) or by the formed malonate radical from [Co^III^(TPP)(CH(CO_2_Me)_2_] (complex **1**) upon light irradiation. This provides an attractive method to replace hazardous diazo compounds or halogenated carbene precursors, such as iodonium ylides, to form cobalt(III) carbene radical intermediates, especially when conditions can be found that allow catalytic turnover. We are confident that regeneration of [Co^III^(TPP)Cl] can unlock catalytic pathways using active methylene compounds as stable and readily available carbene precursors for future reactions, thereby expanding the utilization of radical-type carbene transfer catalysis for the direct functionalization of C–H bonds and olefins.

## Experimental Section

### General Considerations

All experiments were performed under dry and inert (argon) conditions in flame- or oven-dried glassware following standard Schlenk techniques or in a N_2_-filled glovebox unless stated otherwise. All reagents were obtained from commercial suppliers and used without further purification, except for the compounds given below. DCM was predried using a Solvent Purification System (SPS) from MBraun (MB SPS-800, with standard MBraun drying columns). All solvents were dried (further) and stored on activated 3 Å molecular sieves and degassed by sparging with argon or freeze–pump–thaw. Styrene was filtered over basic alumina and sparged with argon before use. A Kessil PR 160L 370 nm (first generation) or 525 nm was used as a light source. ^1^H NMR and ^13^C NMR spectra were recorded on a Bruker DRX 500, AMX 400, or DRX 300 spectrometer at room temperature and referenced to TMS.^25^ Individual peaks are reported as chemical shift (ppm), multiplicity (s: singlet, d: doublet, t: triplet, q: quartet, and m: multiplet), integration, and coupling constant (Hz). During NMR measurements with light irradiation, a Bluepoint 4 with 390–500 nm filter from Honle UV Technology was used with an optic fiber leading to the bottom of the NMR sample. EPR spectra were recorded on a Bruker EMX X-band spectrometer equipped with an ER 4112HV-CF100 He cryostat. UV–vis spectra were recorded on a double beam Shimadzu UV-2600 spectrophotometer in a 1.0 cm quartz cuvette or a 1.0 cm Teflon screw-cap quartz cuvette with an extra 10 mL round-bottom flask, using the solvent as background. Cold spray ionization mass spectrometry (CSI-MS) spectra were collected on an HR-ToF Bruker Daltonik GmbH (Bremen, Germany) Impact II, an ESI-ToF MS capable of resolution of at least 40 000 fwhm, which was coupled to a Bruker cryo-spray unit. The source voltage was between 3 and 6 kV. The sample was introduced with a syringe pump at a flow rate of 180 μL/h. The drying gas (N_2_) was held at −40 °C, and the spray gas was held at −35 °C. The machine was calibrated via direct infusion of a TFA-Na solution. Software acquisition was performed via Compass 2.0 for the Otof series. Field desorption (FD) mass spectra were collected on an AccuTOF GC v 4g, JMS-T100GCV Mass spectrometer (JEOL, Japan).

### DFT Calculations

DFT geometry optimizations were performed without simplifications on full atomic models using TURBOMOLE 7.5.1,^26^ coupled to the PQS Baker optimizer,^27,28^ via the BOpt package.^29^ All calculations were performed in the gas phase with convergence criteria (scfconv = 7) on a m4 grid and Grimme’s version 3 zero-damping dispersion corrections to compensate for the underestimation of metal–ligand interactions from uncorrected DFT calculations.^30^ Geometry optimizations were performed at the ri-DFT BP86^31^/def2-TZVP^32^ level of theory. All minima, without imaginary frequencies, were characterized by calculating the analytical Hessian matrix. Energy output generated in Hartree units was converted to kcal/mol by multiplication with 627.51. Graphical representations of orbitals are obtained using IboView^33^ and visualization of spin densities using IQMol.^34^

### Synthesis of [Co^III^(TPP)Cl]

[Co^III^(TPP)Cl] was synthesized according to a literature procedure.^35^ [Co^II^(TPP)] (300 mg, 0.44 mmol, 1 equiv) was suspended in MeOH (300 mL), and HCl (3 mL, 12 M) was added dropwise. The purple suspension was stirred for 3 h without a stopper on the flask. The red solution was filtered, and the filtrate was concentrated *in vacuo* until a green precipitate was formed. It was filtered and washed with H_2_O (100 mL) and MeOH:H_2_O 1:1 (30 mL). The purple powder was dried over P_2_O_5_ overnight and stored in the glovebox immediately after. Purple powder (92%). ^1^H NMR (300 MHz, CDCl_3_) δ: 8.68 (br s, 8H), 8.26 (br s, 8H), 7.77 (br s, 12H). UV–vis (CH_2_Cl_2_) λ_max_ 405 and 543 nm.

### Synthesis of [Co^III^(TPP)(CH(CO_2_Me)_2_] (Complex 1)

Complex **1** was synthesized by mixing [Co^III^(TPP)Cl] (50 mg, 0.071 mmol, 1 equiv), dimethyl malonate (405 μL, 50 equiv), and KO*t*Bu (450 mg, 60 equiv) in DCM (15 mL, 4.7 mM) and stirred for 15 min. The compound was purified by column chromatography (SiO_2_, cyclohexane/ethyl acetate = 4:1) under dark conditions and stored in an amber vial after concentrating *in vacuo*. Dark red solid (76%). R_f_-value = 0.37. ^1^H NMR (500 MHz, Methylene Chloride-*d*_*2*_) δ 8.92 (s, 8H), 8.20 (br s, 8H), 7.85–7.73 (m, 12H), 1.81 (s, 6H), −3.16 (s, 1H). ^13^C NMR (126 MHz, Methylene Chloride-*d*_*2*_) δ: 171.08, 147.15, 141.80, 133.27, 128.00, 127.11, 123.93, 49.57, −21.43. ^1^H ^13^C-HSQC NMR (500 MHz, Methylene Chloride-*d*_*2*_) δ: −3.16 – −21.43, 1.81–49.57, 7.79–127.11, 8.20–133.27, 8.92–133.27. ^1^H ^13^C-HMBC NMR (500 MHz, Methylene Chloride-*d*_*2*_) δ: −3.16–171.08, 1.81–171.08, 7.79–133.27, 7.79–141.80, 8.92–123.93, 8.92–147.15. HRMS-FD+ (*m*/*z*) calculated for C_49_H_35_CoN_4_O_4_^+^: 802.1990, found: 802.1984 [M^+^]. UV–vis (CH_2_Cl_2_) λ_max_ 312, 375, 413, 525, and 552 nm. Elemental analysis calculated for C_49_H_35_CoN_4_O_4_: C 73.31%, H 4.39%, N 6.98%, Co 7.34%; found: C 73.14%, H 4.31%, N 6.91%, Co 7.28%.

### General Procedure for Cyclopropanation Reaction

To a flame-dried 10 mL Schlenk flask and inside a N_2_-filled glovebox, [Co^III^(TPP)Cl] (10 mol%), KO*t*Bu (2 equiv), benzophenone (1.5 equiv), dimethyl malonate (0.05 mmol, 1 equiv), and styrene (5 equiv) were added. Under an argon flow, dry and degassed DCM (1 mL) was added. The dark red mixture was stirred overnight (16 h) at room temperature (28 °C) and 1000 rpm upon 370 nm (43 W) light irradiation. An external standard solution of 1,3,5-trimethoxybenzene or 1,3,5-tritertbutylbenzene in DCM was added, and the dark red mixture was stirred for a few minutes before concentrating *in vacuo*. The dark red residue was redissolved in DCM-*d*_*2*_ and filtered over cotton when preparing an NMR sample.

### General Procedure for EPR Studies

To a flame-dried 5 mL Schlenk flask, [Co^III^(TPP)Cl] (0.005 mmol, 0.1 equiv), KO*t*Bu (0.11 mmol, 2.2 equiv), and dimethyl malonate (0.05 mmol, 1 equiv) were mixed in toluene-*d*_*8*_. The dark red mixture was freeze–pump–thawed, filtered, and transferred to a quartz EPR tube with benzophenone (0.03 mmol, 0.6 equiv) under an argon flow. The EPR sample was irradiated with 370 nm (43 W) and frozen immediately in liquid nitrogen unless mentioned otherwise.

### General Procedure for NMR Studies

To a flame-dried 10 mL Schlenk flask, complex **1** (0.00137 mmol, 1 equiv), benzophenone (0.002 mmol, 1.5 equiv), and benzene (2 μL) as internal standard were dissolved in CD_2_Cl_2_ (0.5 mL). The dark red solution was freeze–pump–thawed and transferred to a *J*-young NMR tube after filtration. The tube was kept under dark conditions until NMR measurements were performed. All NMR samples were irradiated with a 390–500 nm light source via a light probe attached to the bottom of the NMR machine. To determine the concentration of complex **1**, signals at −3.16 (1H) and 1.82 (6H) ppm were integrated. To determine the concentration of [Co^II^(TPP)], signals at 9.62 (12H) and 12.85 (8H) ppm were integrated.

## Supporting Information Available

Experimental details; synthetic procedures; relevant NMR, EPR, and HRMS data; DFT calculations; Hess cycle used to calculated the R–H BDE in a semiempirical manner (Figure S1); ^1^H NMR spectrum of [Co^III^(TPP)Cl] in CD_2_Cl_2_ (Figure S2); ^1^H NMR spectrum of complex 1 in CD_2_Cl_2_ (Figure S3); ^13^C NMR spectrum of complex 1 in CD_2_Cl_2_ (Figure S4); ^1^H ^13^C HSQC NMR spectrum of complex 1 in CD_2_Cl_2_ (Figure S5); ^1^H ^13^C HMBC NMR spectrum of complex 1 in CD_2_Cl_2_ (Figure S6); HRMS-FD^+^ spectrum of complex 1 (top), zoom-in of HRMS-FD^+^ spectrum (middle), and simulated *m/z* (bottom) (Figure S7); normalized UV–vis absorption spectrum of complex 1 (purple, 21.5 μM) and [Co^III^(TPP)Cl] (orange, 12.2 μM) (Figure S8); crude ^1^H NMR spectrum of the mixture of [Co^II^(TPP)], KO*t*Bu, and dimethyl malonate in CD_2_Cl_2_ (Figure S9); general set-up of photochemical cyclopropanation reactions (Figure S10); general crude ^1^H NMR spectrum of the cyclopropanation reaction under conditions according to entry 1 in Table S1 (Figure S11); control experiments for the cyclopropanation reaction between dimethyl malonate and styrene (Table S1); the cyclopropanation reaction between dimethyl malonate and styrene with oxidants as additives (under aerobic conditions) (Table S2); the cyclopropanation reaction between dimethyl malonate and styrene with deviations deviated reaction conditions (Table S3); the cyclopropanation reaction between dimethyl malonate and different substrates under the optimized reaction conditions (Table S4); UV–vis spectrum of [Co^III^(TPP)Cl] at t = 0 (black line, 12.2 μM) and t = 126 days (blue line, 28.3 μM) in DCM (Figure S12); zoom in of HRMS-CSI^+^ spectra of [Co^III^(TPP)Cl] t = 126 days (top), [Co^III^(TPP)Cl] t = 126 days + 10 μL H_2_O (middle), and simulated spectrum for C_44_H_28_CoN_4_OH^+^ (bottom) (Figure S13); HRMS-CSI^+^ spectrum of [Co^III^(TPP)Cl] at t = 126 days (Figure S14); HRMS-CSI^+^ spectrum of [Co^III^(TPP)Cl] at t = 126 days + 10 μL H_2_O (Figure S15); X-band EPR spectrum obtained upon irradiation (370 nm) of *in situ* formed complex 1 in toluene-*d*_*8*_ mixed with benzophenone at 40 K (Figure S16); X-band EPR spectrum obtained upon irradiation (370 nm) of *in situ* formed complex 1 in toluene-*d*_*8*_ at 40 K (Figure S17); X-band EPR spectrum of *in situ* formed complex 1 in C_6_H_6_ under dark conditions at 20 K (Figure S18); X-band EPR spectrum of [Co^II^(TPP)] in toluene-*d_8_* at 40 K (Figure S19); comparison between the experimental EPR spectra of complex 1 after irradiation (Figure S20); experimental EPR parameters of cobalt(III) carbene radical complex from spectral simulations (Table S5); conversion of complex 1 under dark conditions (blue, 2.75 mM) and upon 390–500 nm light irradiation (purple, 2.68 mM) in DCM-*d*_*2*_ (Figure S21); (a) conversion of complex 1 and (b) formation of [Co^II^(TPP)] with benzophenone (orange) and without benzophenone (purple) upon 390-500 nm light irradiation (Figure S22); UV–vis spectrum of complex 1 (21.5 μM) in DCM under dark conditions (Figure S23); graphical representation of all optimized structures and Mulliken spin density plots of all optimized structures (Table S6); negative frequencies and energies (Hartree) of all optimized structures at 298 K (Table S7) (PDF)
A text file of all computed molecule Cartesian coordinates in xyz format (XYZ)

## Supplementary Material

om4c00127_si_001.pdf

om4c00127_si_002.xyz

## Notes

A preprint version of this manuscript was uploaded to *ChemRxiv*.^36^
