# Supplementary material for: Light Induced Cobalt(III) Carbene Radical Formation from Dimethyl Malonate As Carbene Precursor
Source: Organometallics. 2024 May 24;43(11):1299–307. doi: 10.1021/acs.organomet.4c00127 (PMC11167645; doi:10.1021/acs.organomet.4c00127)
Supplement: Supplementary file 1 — om4c00127_si_001.pdf [file om4c00127_si_001.pdf]

# Supplementary Information

## Light Induced Cobalt(III) Carbene Radical Formation from Dimethyl Malonate as Carbene Precursor

Demi D. Snabilié, Rens Ham, Joost N. H. Reek and Bas de Bruin*

*Van 't Hoff Institute for Molecular Sciences, University of Amsterdam, Science Park 904, 1098 XH Amsterdam, The Netherlands*

**1. General considerations** ........ 1
**2. Synthesis and characterization of compounds** ........ 3
**3. Cyclopropanation reactions** ........ 9
**4. EPR studies** ........ 16
**5. NMR studies** ........ 20
**6. UV-Vis studies** ........ 22
**7. Computational details** ........ 23
**8. References** ........ 25

## 1. General considerations

All experiments were performed under dry and inert (Argon) conditions in flame- or oven-dried glassware following standard Schlenk techniques or in a $N_2$-filled glovebox, unless stated otherwise. All reagents were obtained from commercial suppliers and used without further purification, except for the compounds given below. DCM was pre-dried using a Solvent Purification System (SPS) from MBraun (MB SPS-800, with standard MBraun drying columns). All solvents were dried (further) and stored on activated 3 Å molecular sieves and degassed by sparging with argon or freeze-pump-thaw. Styrene was filtered over basic alumina and sparged with Argon before use. A Kessil PR 160L 370 nm (first generation) or 525 nm were used as light sources. $^1H$ NMR and $^{13}C$ NMR spectra were recorded on a Bruker DRX 500, AMX 400, or DRX 300 spectrometer at room temperature and referenced to TMS.[1] Individual peaks are reported as: chemical shift (ppm), multiplicity (s: singlet, d: doublet, t: triplet, q: quartet, m: multiplet), integration, coupling constant (Hz). During NMR measurements with light irradiation, a Bluepoint 4 with 390-500 nm filter from Honle UV Technology was used with an optic fiber leading to the bottom of the NMR sample. EPR spectra were recorded on a Bruker EMX X-band spectrometer equipped with an ER 4112HV-CF100 He cryostat. UV-Vis spectra were recorded on a double beam Shimadzu UV-2600 spectrophotometer in a 1.0 cm quartz cuvette or a 1.0 cm Teflon screw-cap quartz cuvette with an extra 10 mL round bottom flask, using the solvent as background. Cold spray ionization mass spectrometry (CSI-MS) spectra were collected on a HR-ToF Bruker Daltonik GmbH (Bremen, Germany) Impact II, an ESI-ToF MS capable of resolution of at least 40000 FWHM, which was coupled to a Bruker cryo-spray unit. The source voltage was between 3 and 6 kV. The sample was introduced with a syringe pump at a flow rate of 180 µl/hr. The drying gas ($N_2$) was held at -40°C and the spray gas was held at -35°C. The machine was calibrated via direct infusion of a TFA-Na solution. Software acquisition Compass 2.0 for Otof series. Field desorption (FD) mass spectra were collected on an AccuTOF GC v 4g, JMS-T100GCV Mass spectrometer (JEOL, Japan).

*DFT calculations*

DFT geometry optimizations were performed without simplifications on full atomic models using TURBOMOLE 7.5.1,[2] coupled to the PQS Baker optimizer,[3, 4] via the BOpt package.[5] All calculations were performed in the gas phase with convergence criteria (scfconv = 7) on a m4 grid and Grimme's version 3 zero-damping dispersion corrections to compensate for the underestimation of metal-ligand interactions from uncorrected DFT calculations.[6] For a description of the functional and basis sets used, see below. All minima, without imaginary frequencies, were characterized by calculating the analytical Hessian matrix. Energy output generated in Hartree units was converted to kcal/mol by multiplication with 627.51. Graphical representations of orbitals are obtained using IboView[7] and visualization of spin densities using IQMol.[8]

*Calculation of the bond dissociation energies (BDEs)*

1) All input structures were generated as protein database files (.pdb).
2) A geometry optimization was performed at the ri-DFT BP86[9]/def2- TZVP[10] level of theory.
3) Depending on the bond, the BDE was calculated via the following calculations:
   a. R–X bond:

      $$BDE = E_{R\cdot} + E_{X\cdot} - E_{R-X}$$

   b. R–H bond: The DFT calculated BDEs were calculated using a semiempirical Hess cycle, as shown in Figure S1.[11, 12]

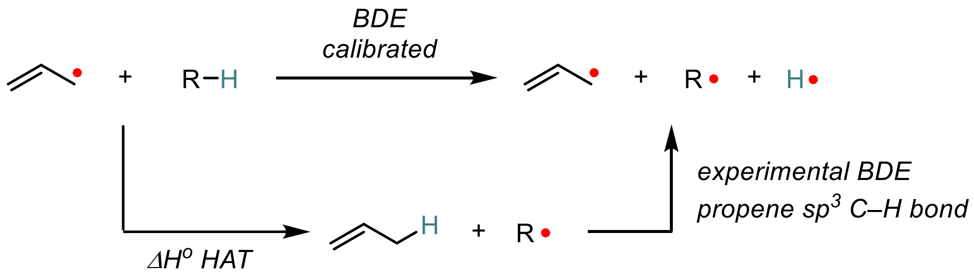


Figure S1. Hess cycle used to calculated the R-H BDE in a semi-empirical manner.

$BDE_{propene}$[13] = +88.8 kcal/mol (experimental BDE propene *sp*$^3$ C–H bond)

$\Delta H^{o}\ HAT = E_{R\cdot} + E_{propene}{}^{13} - E_{R-H} - E_{propene\cdot}{}^{13}$

$BDE_{calibrated} = BDE_{propene} + \Delta H^{o}\ HAT$

## 2. Synthesis and characterization of compounds

### Synthesis of [Co$^{III}$(TPP)Cl]

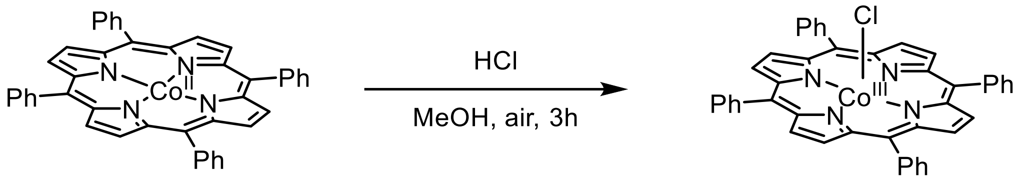


[Co$^{II}$(TPP)] [Co$^{III}$(TPP)Cl]

[Co$^{III}$(TPP)Cl] was synthesized according to a literature procedure.[14] [Co$^{II}$(TPP)] (300 mg, 0.44 mmol, 1 eq) was suspended in MeOH (300 mL) and HCl (3 mL, 12 M) was added dropwise. The purple suspension was stirred for 3 hours without a stopper on the flask. The red solution was filtered and the filtrate was concentrated *in vacuo* until a green precipitate was formed. It was filtered and washed with $H_2O$ (100 mL) and MeOH:$H_2O$ 1:1 (30 mL). The purple powder was dried over $P_2O_5$ overnight and stored in the glovebox immediately after. Purple powder (92%).

*Note: it is important to store the obtained [Co$^{III}$(TPP)Cl] under dry conditions immediately after synthesis to avoid hydration and thus deactivation (see section 3.3).*

$^1$H NMR (300 MHz, $CDCl_3$) δ 8.68 (br s, 8H), 8.26 (br s, 8H), 7.77 (br s, 12H). (Figure S2).

UV-Vis ($CH_2Cl_2$) $\lambda_{max}$ 405 nm and 543 nm (Figure S8).

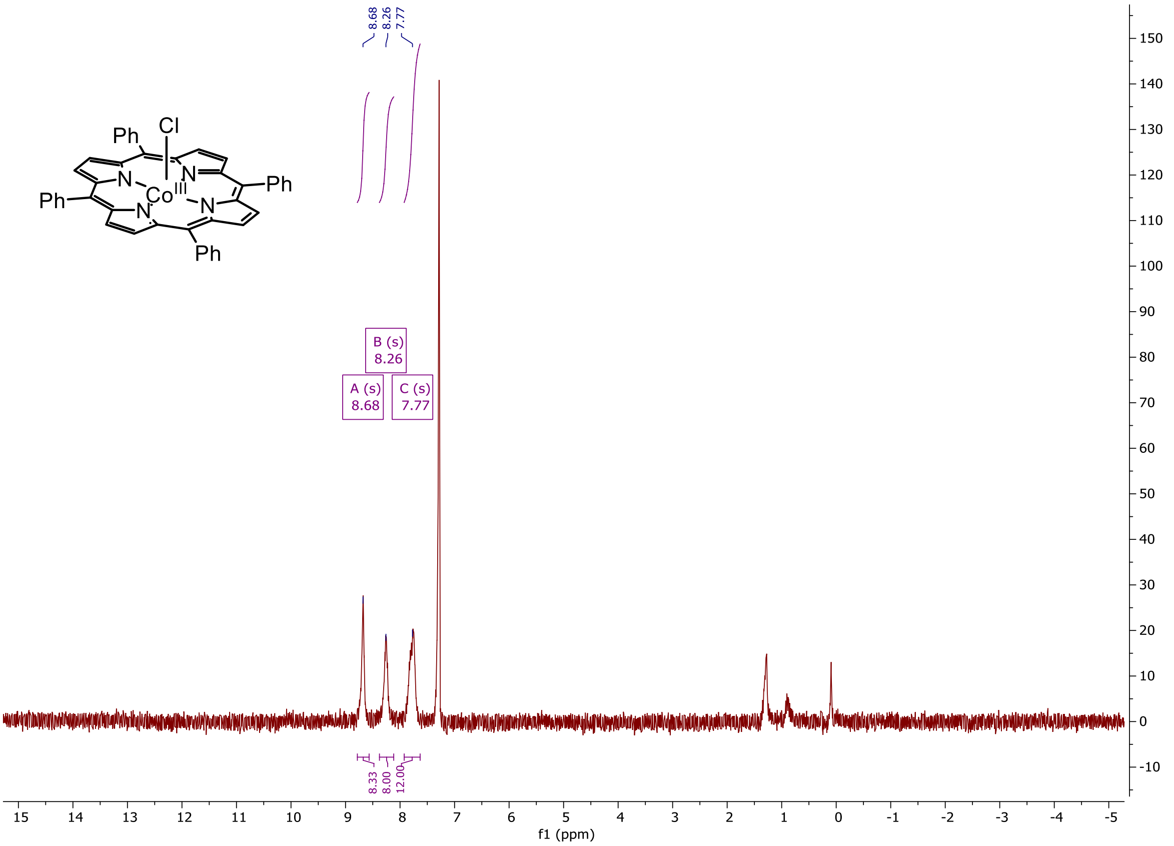


Figure S2. $^1$H NMR spectrum of [Co$^{III}$(TPP)Cl] in $CD_2Cl_2$.

**Synthesis of [Co$^{III}$(TPP)(CH(CO$_2$Me)$_2$]** (Complex **1**)

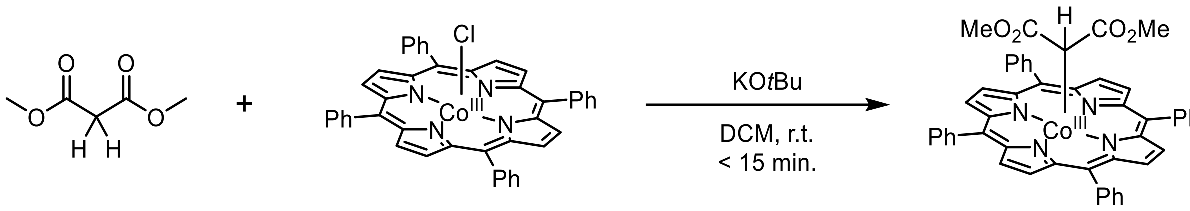


Complex **1** was synthesized by mixing [Co$^{III}$(TPP)Cl] (50 mg, 0.071 mmol, 1 eq), dimethyl malonate (405 µL, 50 eq.) and KO*t*Bu (450 mg, 60 eq.) in DCM (15 mL, 4.7 mM) and stirred for 15 minutes. The compound was purified by column chromatography ($SiO_2$, cyclohexane/ethyl acetate = 4:1) under dark conditions and stored in an amber vial after concentrating *in vacuo*. Dark red solid (76%). $R_f$-value = 0.37.

*Note: this compound is light sensitive.*

$^1$H NMR (500 MHz, Methylene Chloride-*d*$_2$) δ 8.92 (s, 8H$_E$), 8.20 (br s, 8H$_D$), 7.85 – 7.73 (m, 12H$_C$), 1.81 (s, 6H$_B$), -3.16 (s, 1H$_A$) (Figure S3).

$^{13}$C NMR (126 MHz, Methylene Chloride-*d*$_2$) δ 171.08 (C$_c$), 147.15 (C$_k$), 141.80 (C$_j$), 133.27 (C$_{d,e,i}$), 128.00 (C$_g$), 127.11 (C$_h$), 123.93 (C$_f$), 49.57 (C$_b$), -21.43 (C$_a$) (Figure S4).

$^1$H $^{13}$C-HSQC NMR (500 MHz, Methylene Chloride-*d*$_2$) δ -3.16 – -21.43, 1.81 – 49.57, 7.79 – 127.11, 8.20 – 133.27, 8.92 – 133.27 (Figure S5).

$^1$H $^{13}$C-HMBC NMR (500 MHz, Methylene Chloride-*d*$_2$) δ -3.16 – 171.08, 1.81 – 171.08, 7.79 – 133.27, 7.79 – 141.80, 8.92 – 123.93, 8.92 – 147.15 (Figure S6).

HRMS-FD$^+$ (*m/z*) calculated for $C_{49}H_{35}CoN_4O_4^+$: 802.1990, found: 802.1984 [M$^+$] (Figure S7).

UV-Vis ($CH_2Cl_2$) $\lambda_{max}$ 312 nm, 375 nm, 413 nm, 525 nm, and 552 nm (Figure S8).

Elemental analysis calculated for $C_{49}H_{35}CoN_4O_4$: C 73.31%, H 4.39%, N 6.98%, O 7.97%. Co 7.34%; found: C 73.14%, H 4.31%, N 6.91%, O 8.36%, Co 7.28%.

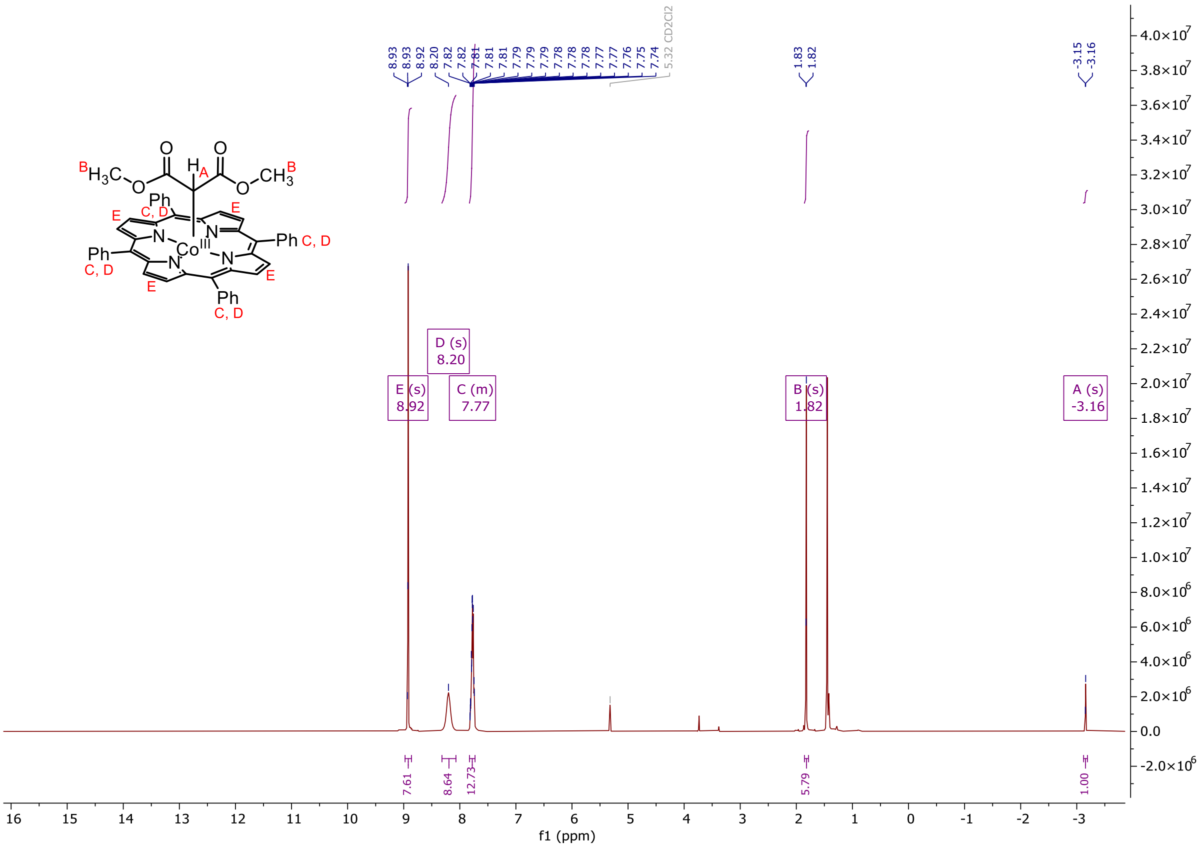


Figure S3. $^{1}H$ NMR spectrum of complex **1** in $CD_2Cl_2$.

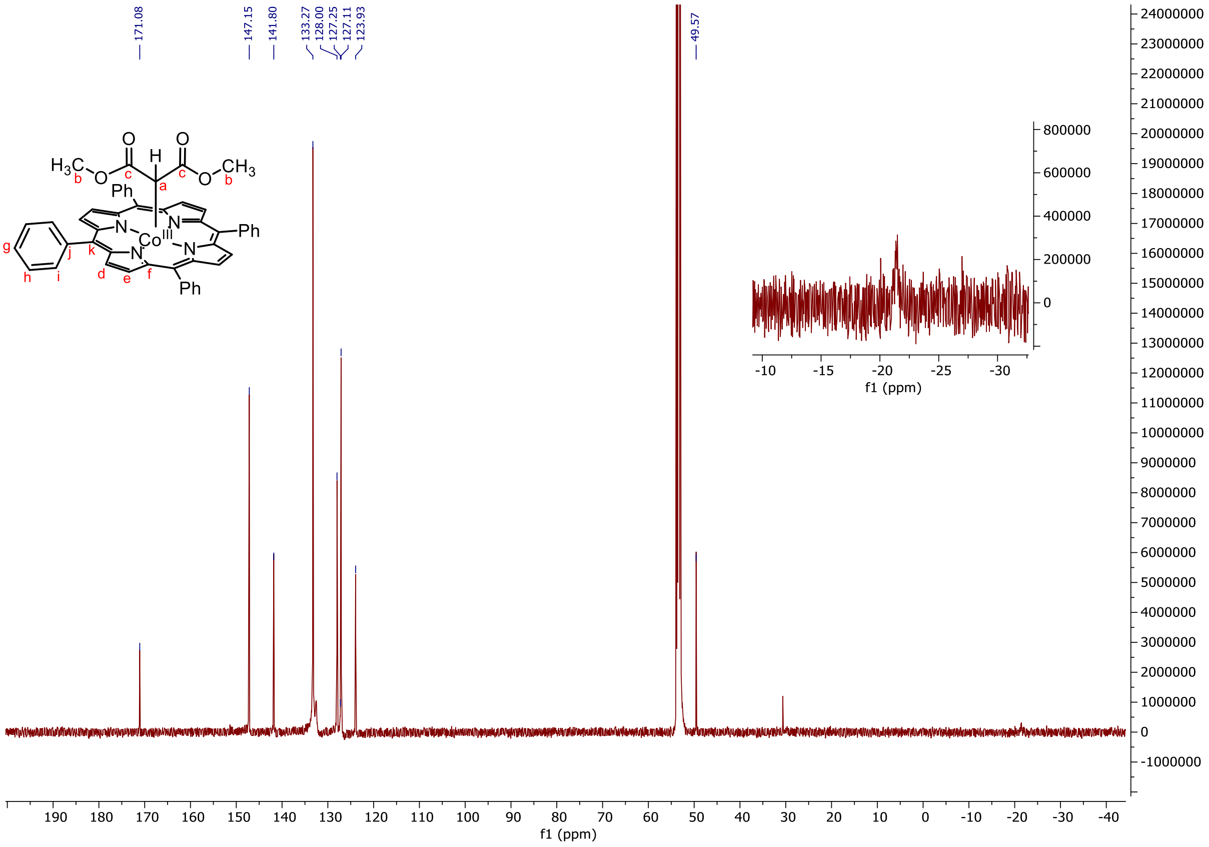


Figure S4. $^{13}C$ NMR spectrum of complex **1** in $CD_2Cl_2$.

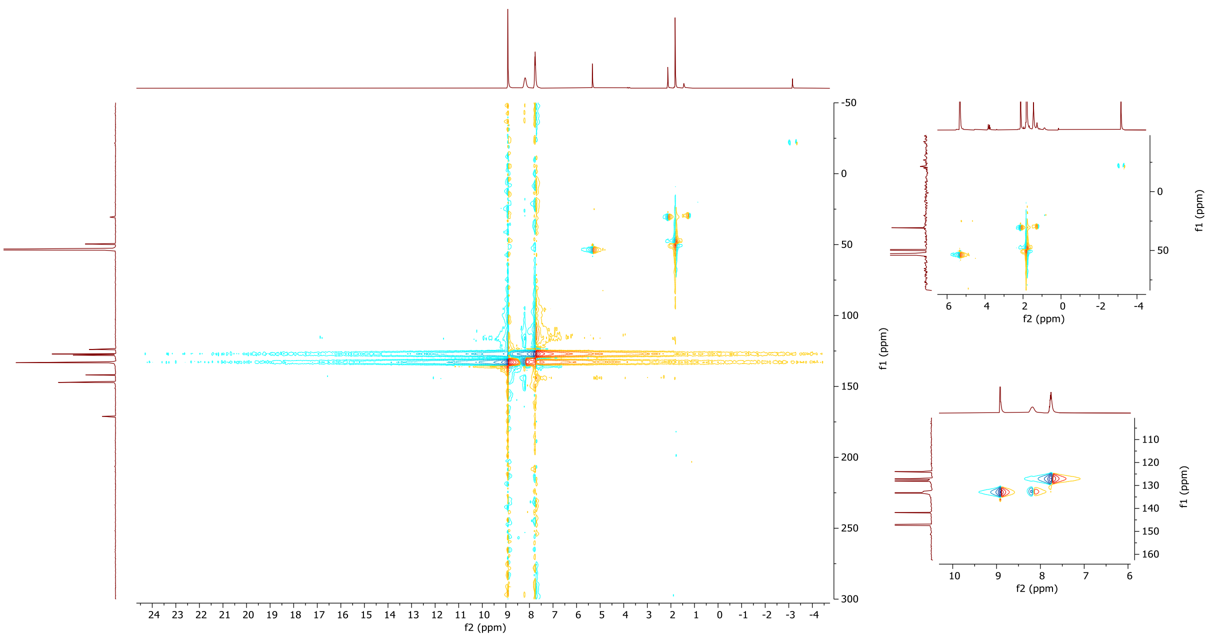


Figure S5. $^{1}$H $^{13}$C HSQC NMR spectrum of complex **1** in $CD_2Cl_2$.

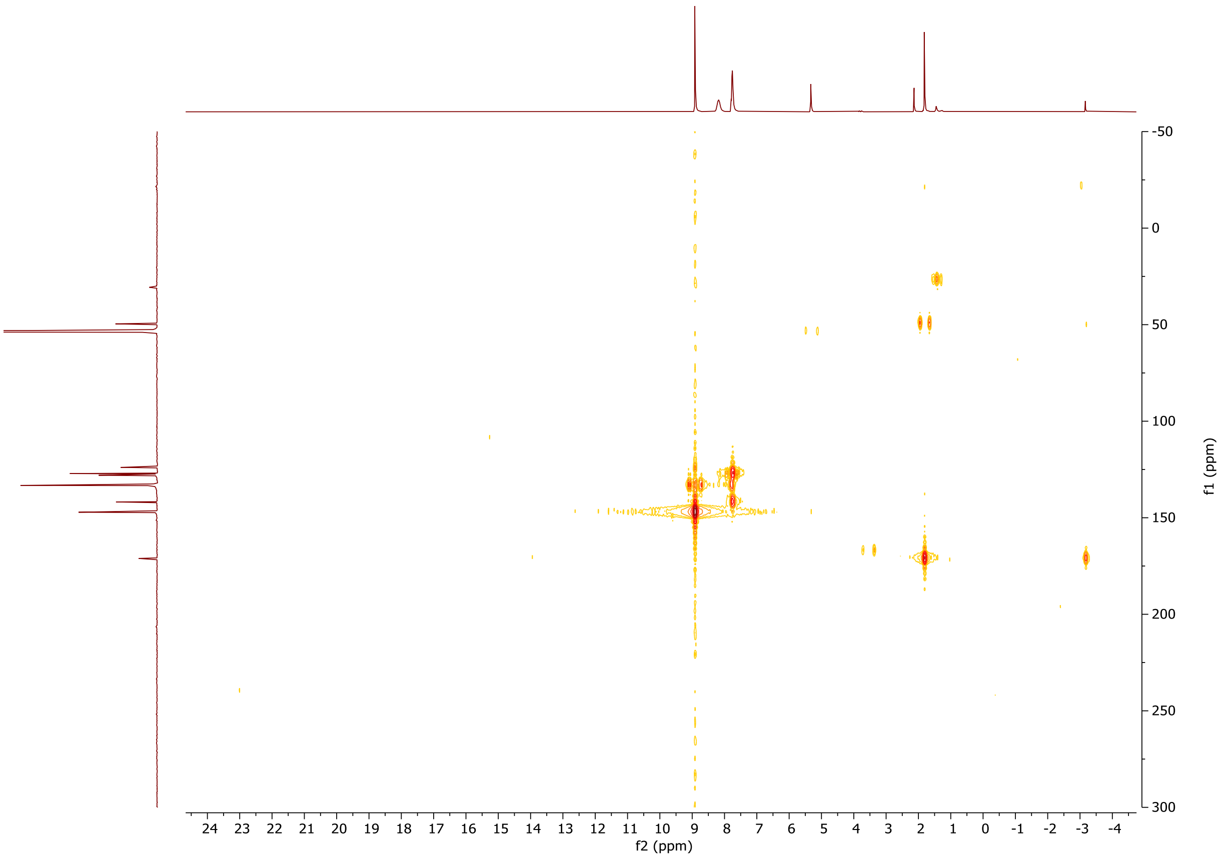


Figure S6. $^{1}$H $^{13}$C HMBC NMR spectrum of complex **1** in $CD_2Cl_2$.

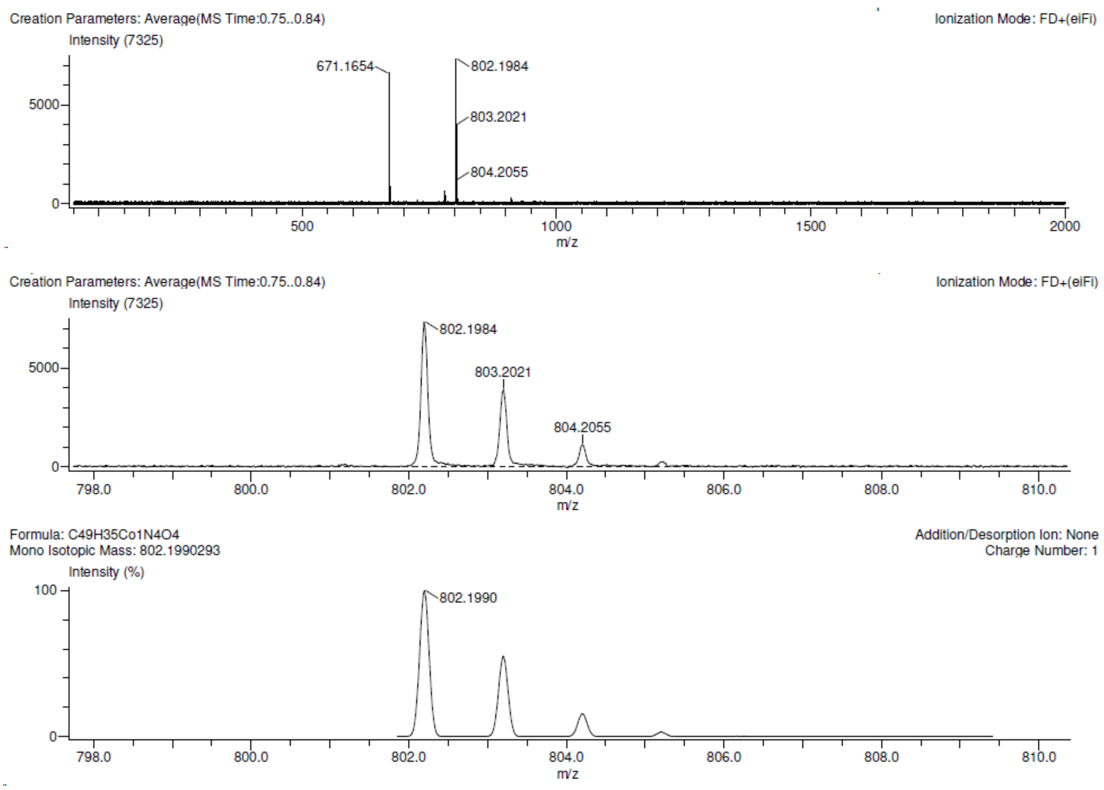


Figure S7. HRMS-FD$^+$ spectrum of complex **1** (top), zoom-in of HRMS-FD$^+$ spectrum (middle) and simulated *m/z* (bottom). HRMS-FD$^+$ (*m/z*) calculated for $C_{49}H_{35}CoN_4O_4^+$: 802.1990, found: 802.1984 [M$^+$].

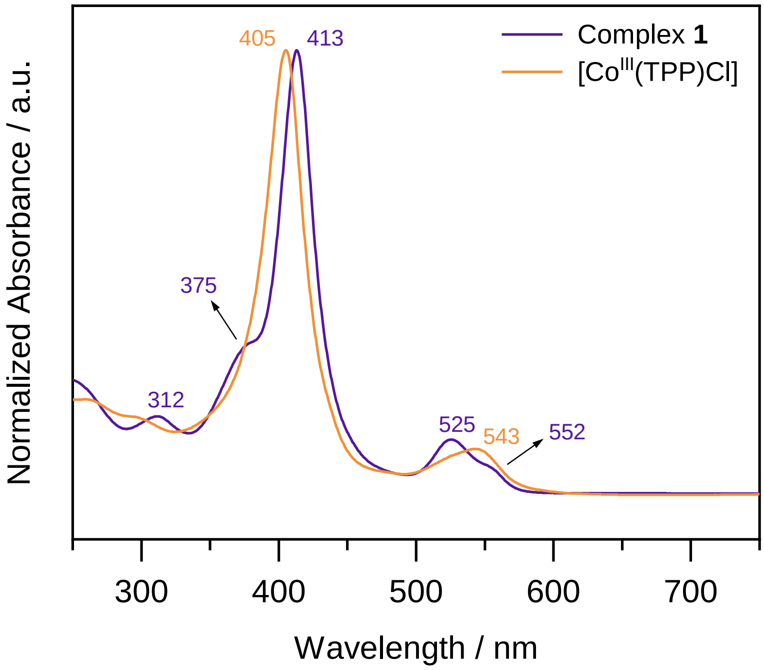


Figure S8. Normalized UV-Vis absorption spectrum of complex **1** (purple, 21.5 µM) and [Co$^{III}$(TPP)Cl] (orange, 12.2 µM).

**Interaction between dimethyl malonate and [Co$^{II}$(TPP)]**

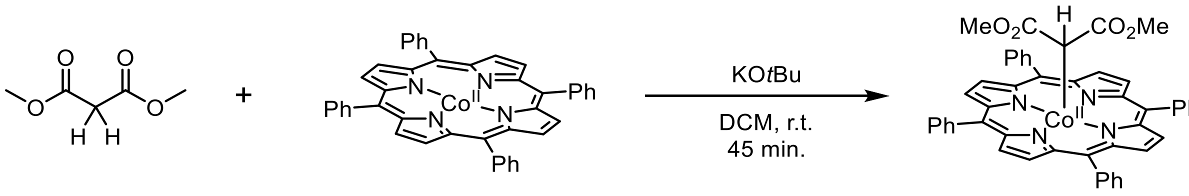


*not observed*

[Co$^{II}$(TPP)] (3.4 mg, 0.005 mmol, 1 eq), dimethyl malonate (20 µL, 0.175 mmol, 35 eq.) and KO*t*Bu (23 mg, 0.20 mmol, 40 eq.) were mixed in $CD_2Cl_2$ (1 mL) and stirred for 45 minutes. The NMR sample was filtered over a syringe filter before $^1H$ NMR was measured.

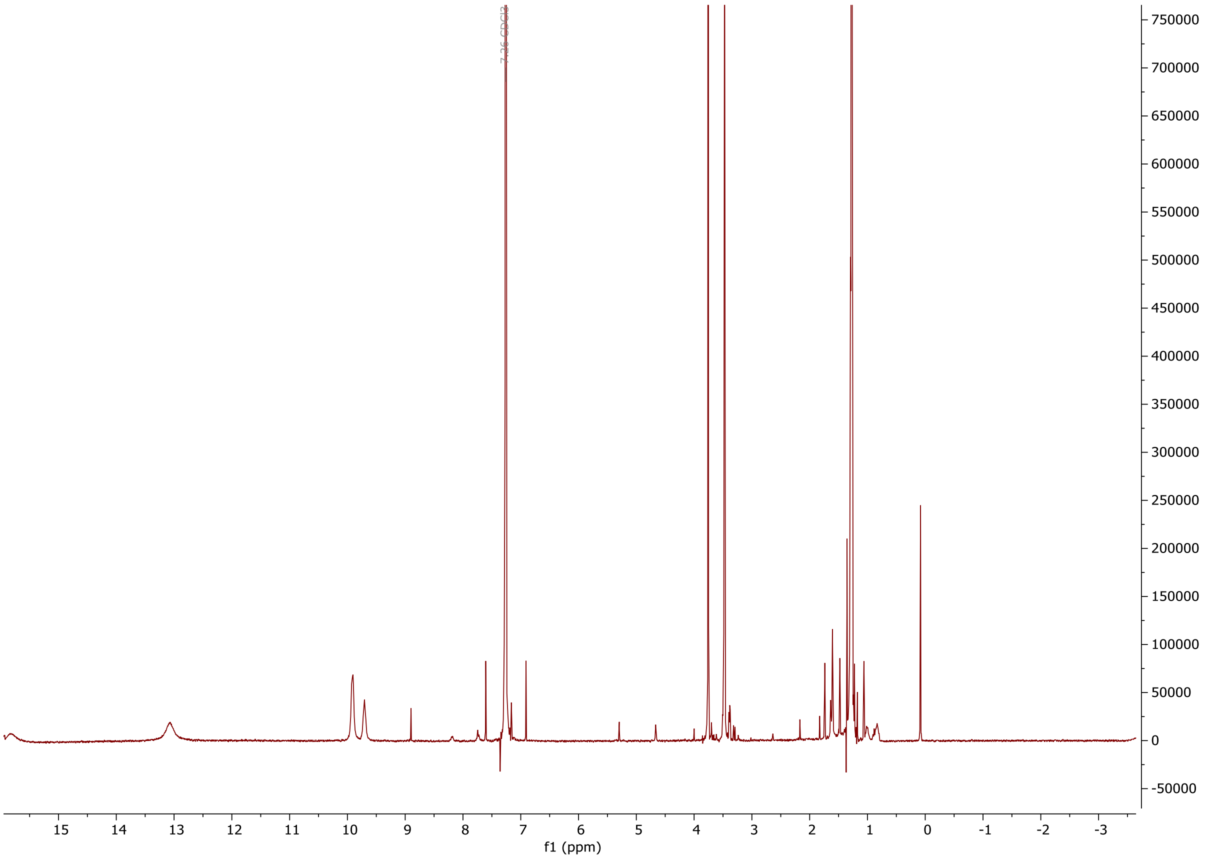


Figure S9. Crude $^1H$ NMR spectrum of the mixture of [Co$^{II}$(TPP)], KO*t*Bu, and dimethyl malonate in $CD_2Cl_2$. No signal at -3.16 ppm was observed.

## 3. Cyclopropanation reactions

### *3.1 General procedure cyclopropanation reaction:*

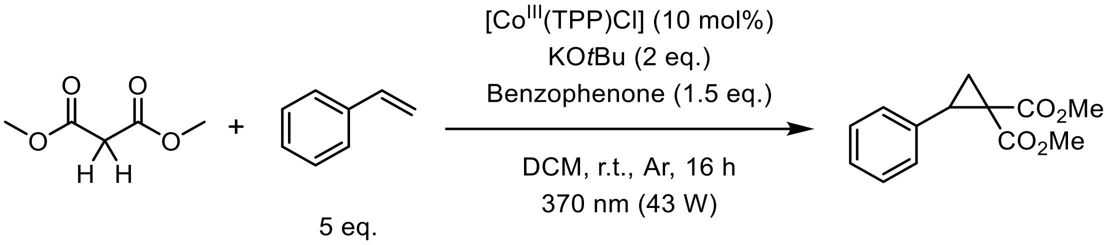


To a flame-dried 10 mL Schlenk flask and inside a $N_2$-filled glovebox, [$Co^{III}$(TPP)Cl] (10 mol%), KO*t*Bu (2 eq.), benzophenone (1.5 eq.), dimethyl malonate (0.05 mmol, 1 eq.) and styrene (5 eq.) were added. Under an Argon flow, dry and degassed DCM (1 mL) was added. The dark red mixture was stirred overnight (16 h) at room temperature (±28ºC) at 1000 rpm upon 370 nm (43 W) light irradiation (see Figure S10). An external standard solution of 1,3,5-trimethoxybenzene or 1,3,5-tritertbutylbenzene in DCM was added and the dark red mixture was stirred for a few minutes, before concentrating *in vacuo*. The dark red residue was redissolved in DCM-$d_2$ and filtered over cotton when preparing a NMR sample.

*Note: it is important to work under completely dry conditions to avoid inactivity of the reaction, including storage of the starting materials (see section 3.3).*

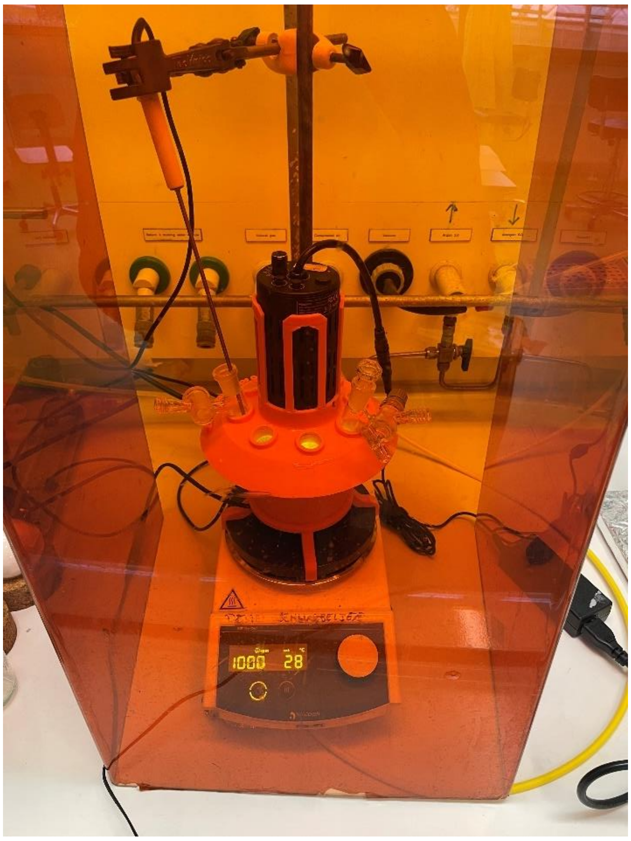


Figure S10. General set-up of photochemical cyclopropanation reactions.

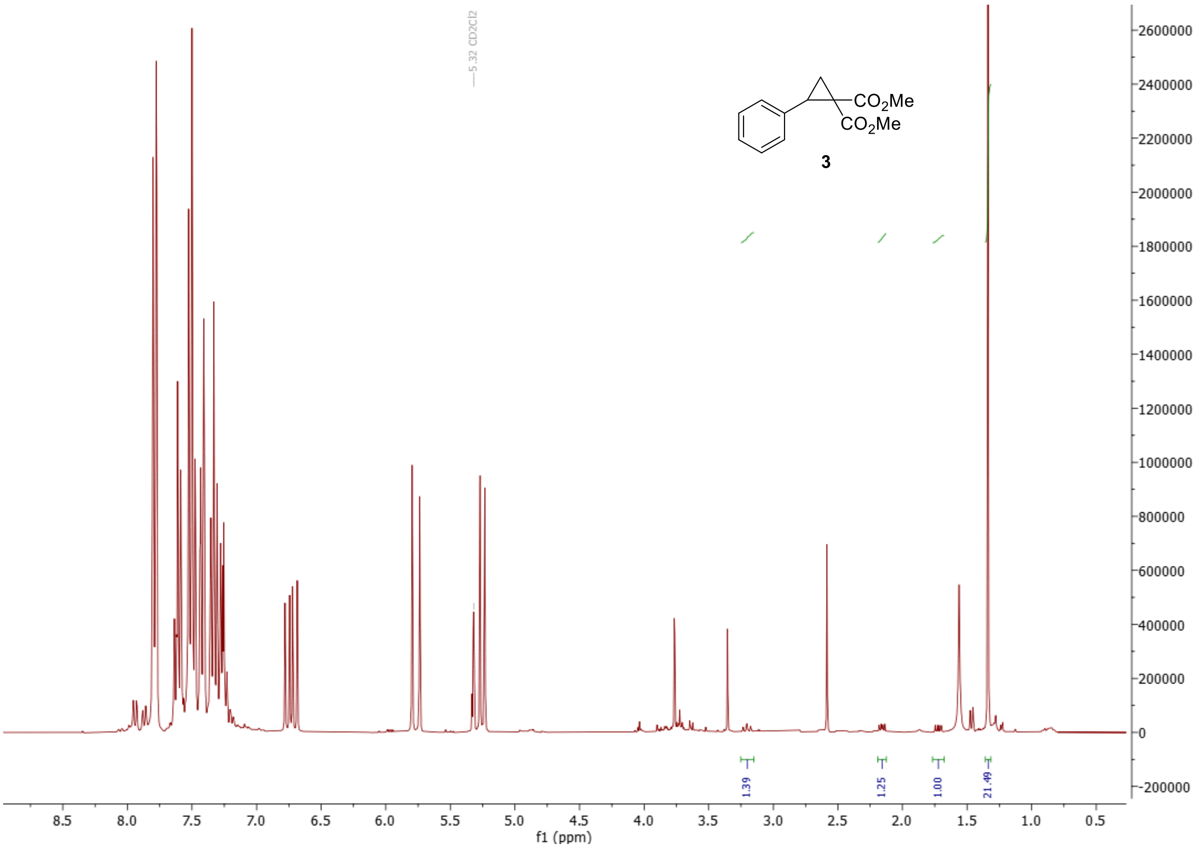


Figure S11. General crude $^{1}$H NMR spectrum of the cyclopropanation reaction under conditions according to entry 1 in Table S1. 1,3,5-tri-*tert*butylbenzene was added as an external standard.

### *3.2 Control experiments:*

**Table S1. Control experiments for the cyclopropanation reaction between dimethyl malonate and styrene.[a]**

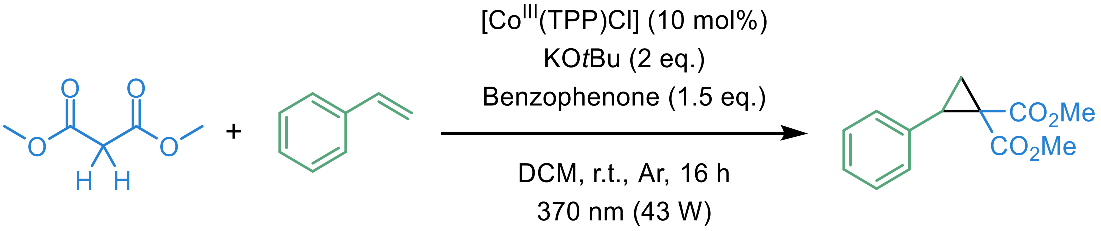


| **Entry** | **Deviations** | **Yield** |
|---|---|---|
| **1** | None | 11% ± 2%[b] |
| **2** | no [Co$^{III}$(TPP)Cl] | 0% |
| **3** | no KO*t*Bu | 0% |
| **4** | no benzophenone | <5% |
| **5** | Dark | 0% |
| **6** | [Co$^{II}$(TPP)] | 0% |
| **7** | no [Co$^{III}$(TPP)Cl], no KO*t*Bu | 0% |
| **8** | no KO*t*Bu, no benzophenone | 0% |

[a]Conditions: dimethyl malonate (0.05 mmol, 1 eq.), [Co] (10 mol%), styrene (5 eq.), benzophenone (1.5 eq.), and KO*t*Bu (2 eq.) were mixed in dry DCM (1 mL) under argon and stirred overnight at 28°C upon light irradiation of 370 nm (43 W). Yields are based on $^1$H NMR analysis of the crude products with 1,3,5-tritertbutylbenzene or 1,3,5-trimethoxybenzene as an external standard. [b]Yield is an average of 6 experiments.

### *3.3 Oxidation experiments:*

**Table S2. The cyclopropanation reaction between dimethyl malonate and styrene with oxidants as additives (under aerobic conditions).[a]**

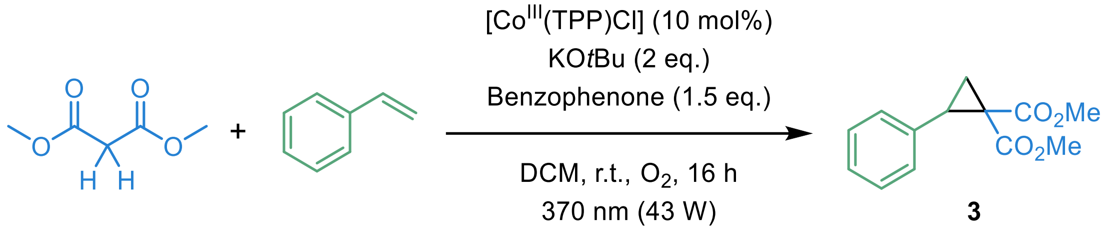


| Entry | Additives | Yield |
|---|---|---|
| **1** | air | 12% |
| **2** | Chloranil (1.2 eq.) | 0% |
| **3** | DDQ (1.2 eq.) | 0% |
| **4** | $K_2S_2O_8$ (1.2 eq.) | 0% |

[a]Conditions: dimethyl malonate (0.05 mmol, 1 eq.), [Co$^{III}$(TPP)Cl] (10 mol%), styrene (5 eq.), benzophenone (1.5 eq.), KO*t*Bu (2 eq.), and oxidant (1.2 eq.) were mixed in dry DCM (1 mL) under aerobic conditions and stirred overnight at 28°C upon light irradiation of 370 nm (43 W). Yields are based on $^1$H NMR analysis of the crude products with 1,3,5-tritertbutylbenzene or 1,3,5-trimethoxybenzene as an external standard. DDQ = 2,3-Dichloro-5,6-dicyano-p-benzoquinone.

### *3.4 Reaction optimization:*

**Table S3. The cyclopropanation reaction between dimethyl malonate and styrene with deviations deviated reaction conditions.[a]**

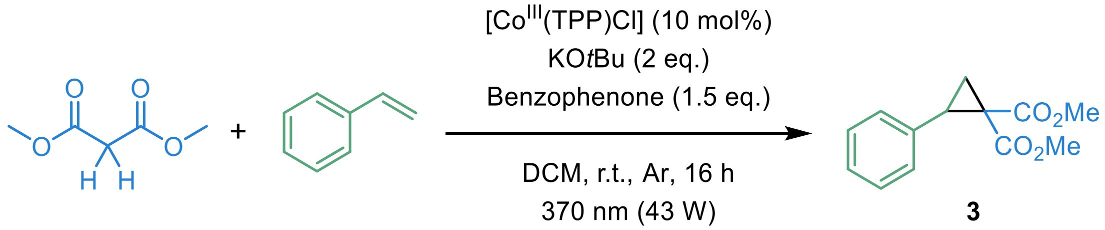


| Entry | Deviations | Yield |
|---|---|---|
| **1** | None | 11% ± 2%[b] |
| **2** | 5 mol% [Co$^{III}$(TPP)Cl] | 7% |
| **3** | 15 mol% [Co$^{III}$(TPP)Cl] | 12% |
| **4** | 20 mol% [Co$^{III}$(TPP)Cl] | Traces |
| **5** | $C_6H_6$ instead of DCM | Traces |
| **6** | MeCN instead of DCM | 0% |
| **7** | $Et_3N$ instead of KO*t*Bu | 0% |
| **8** | DBU instead of KO*t*Bu | 0% |

| | | |
|---|---|---|
| **9** | $Cs_2CO_3$ instead of KO*t*Bu | 0% |

[a]Conditions: dimethyl malonate (0.05 mmol, 1 eq.), [Co$^{III}$(TPP)Cl] (5/10/15/20 mol%), styrene (5 eq.), benzophenone (1.5 eq.), and KO*t*Bu/$Et_3N$/DBU/$Cs_2CO_3$ (2 eq.) were mixed in dry DCM/$C_6H_6$/MeCN (1 mL) under argon and stirred overnight at 28°C upon light irradiation of 370 nm (43 W). Yields are based on $^1$H NMR analysis of the crude products with 1,3,5-tritertbutylbenzene or 1,3,5-trimethoxybenzene as an external standard. DBU = 1,8-Diazabicyclo[5.4.0]undec-7-ene. [b]Yield is an average of 6 experiments.

### *3.5 Exploration of different substrates:*

**Table S4. The cyclopropanation reaction between dimethyl malonate and different substrates under the optimized reaction conditions.[a]**

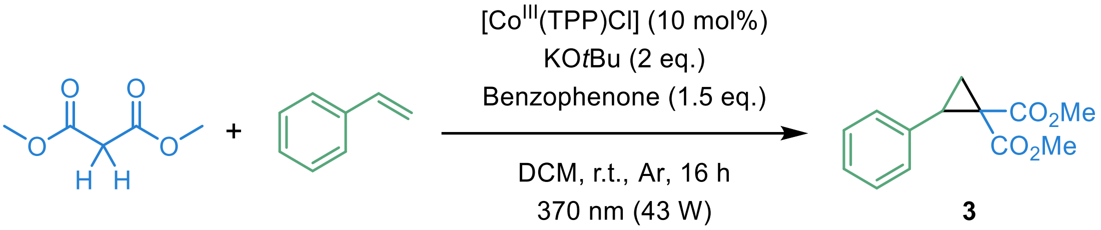


| Entry | Substrate | Yield |
|---|---|---|
| **1** | Styrene | 11% ± 2%[b] |
| **2** | Ethylbenzene | 0% |
| **3** | Thioanisole | 0% |
| **4** | Cyclopentene | 0% |
| **5** | Phenylacetylene | 0% |

[a]Conditions: dimethyl malonate (0.05 mmol, 1 eq.), [Co$^{III}$(TPP)Cl] (10 mol%), substrate (5 eq.), benzophenone (1.5 eq.), and KO*t*Bu (2 eq.) were mixed in dry DCM (1 mL) under argon and stirred overnight at 28°C upon light irradiation of 370 nm (43 W). Yields are based on $^1$H NMR analysis of the crude products with 1,3,5-tritertbutylbenzene or 1,3,5-trimethoxybenzene as an external standard. [b]Yield is an average of 6 experiments.

### *3.6 Moisture affecting [Co$^{III}$(TPP)Cl]:*

During investigation of the cyclopropanation reaction, we occasionally encountered inactivity when the reaction was performed according to the conditions of entry 1 in Table S1. We hypothesized that moisture affects [Co$^{III}$(TPP)Cl] over time, leading to inactivity during the cyclopropanation reaction. UV-Vis and HRMS spectroscopy was used to determine changes in the structure of [Co$^{III}$(TPP)Cl] (Figure S12). When the UV-Vis spectrum was measured immediately after [Co$^{III}$(TPP)Cl] was synthesized, a Soret band at 405 nm and a Q-band at 543 nm was observed (Figure 12a, black line). After 126 days, the UV-Vis spectrum was measured again (blue line). A small change around 440 nm can be observed when comparing the two spectra. Upon addition of an excess of $H_2O$, a shoulder was observed at the same wavelength (440 nm) (Figure S12b). HRMS measurements confirmed our hypothesis of moisture affecting the cobalt complex (Figure S12c). In the presence of an excess of $H_2O$, a signal at 688.1713 m/z was observed that corresponds to [Co$^{III}$(TPP)OH]. The same signal, although significantly less intense, was observed when no $H_2O$ was added to the sample. This result demonstrates that a small amount of water changes the composition of the cobalt metal complex. When newly synthesized [Co$^{III}$(TPP)Cl] was dried over $P_2O_5$ and the reaction was prepared in the glovebox, cyclopropane **3** could be observed again in 10% yield (based on $^1$H NMR analysis).

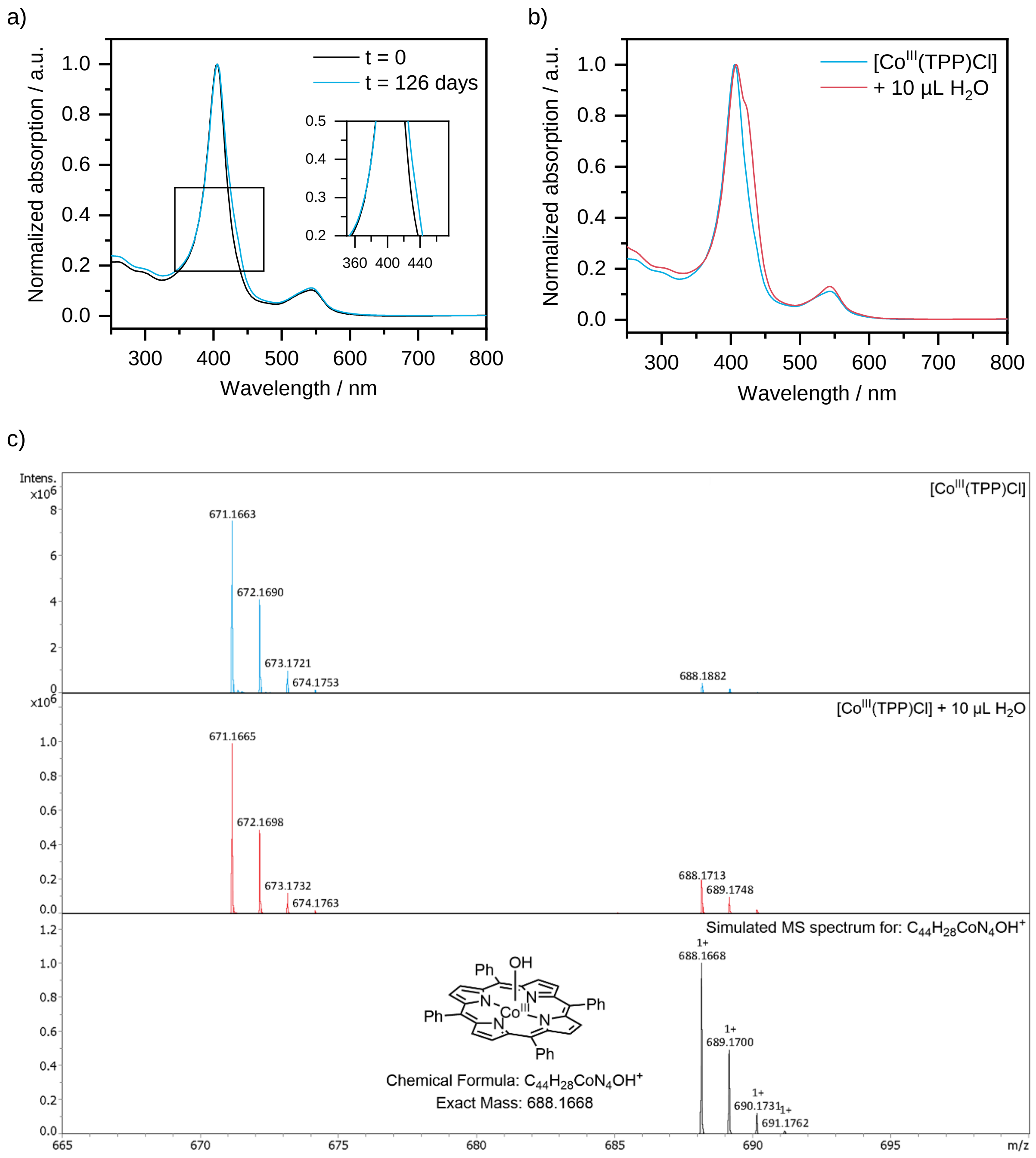


Figure S12. a) UV-Vis spectrum of [Co$^{III}$(TPP)Cl] at t = 0 (black line, 12.2 µM) and t = 126 days (blue line, 28.3 µM) in DCM. b) Addition of 10 µL $H_2O$ (1965 eq.) to [Co$^{III}$(TPP)Cl] solution (28.3 µM) in DCM. c) HRMS-CSI$^+$ spectra of [Co$^{III}$(TPP)Cl] *t*= 126 days (top), [Co$^{III}$(TPP)Cl] *t* = 126 days + 10 µL $H_2O$ (middle), and simulated spectrum for $C_{44}H_{28}CoN_4OH^+$ (bottom).

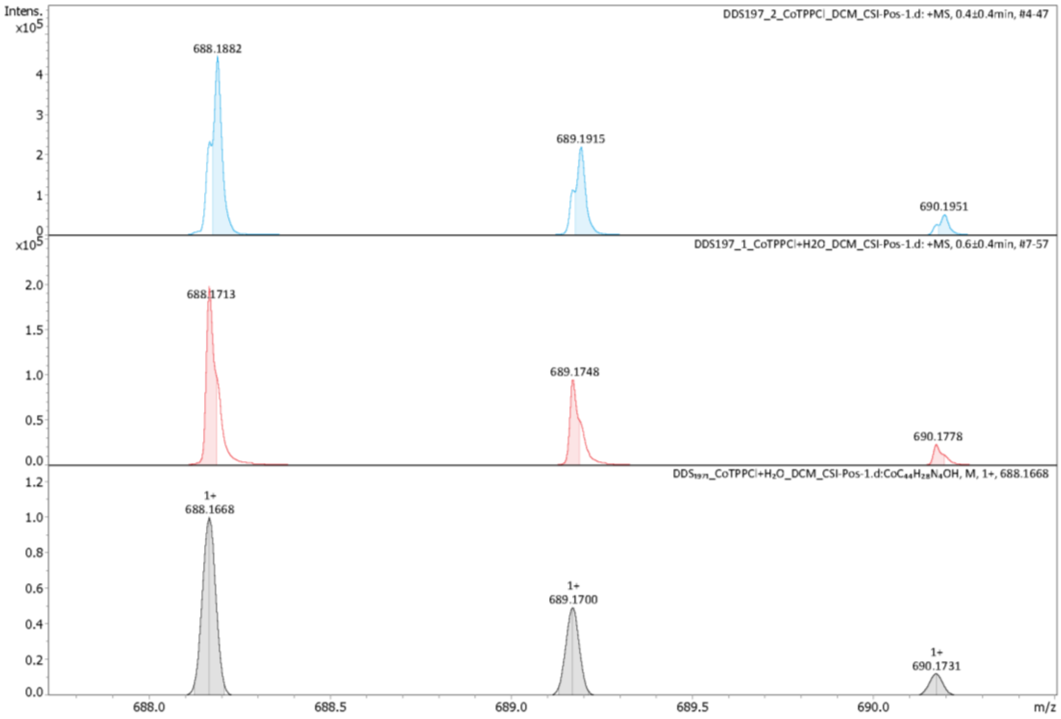


Figure S13. Zoom in of HRMS-CSI+ spectra of [Co$^{III}$(TPP)Cl] t = 126 days (top), [Co$^{III}$(TPP)Cl] t = 126 days + 10 µL $H_2O$ (middle), and simulated spectrum for $C_{44}H_{28}CoN_4OH^+$ (bottom).

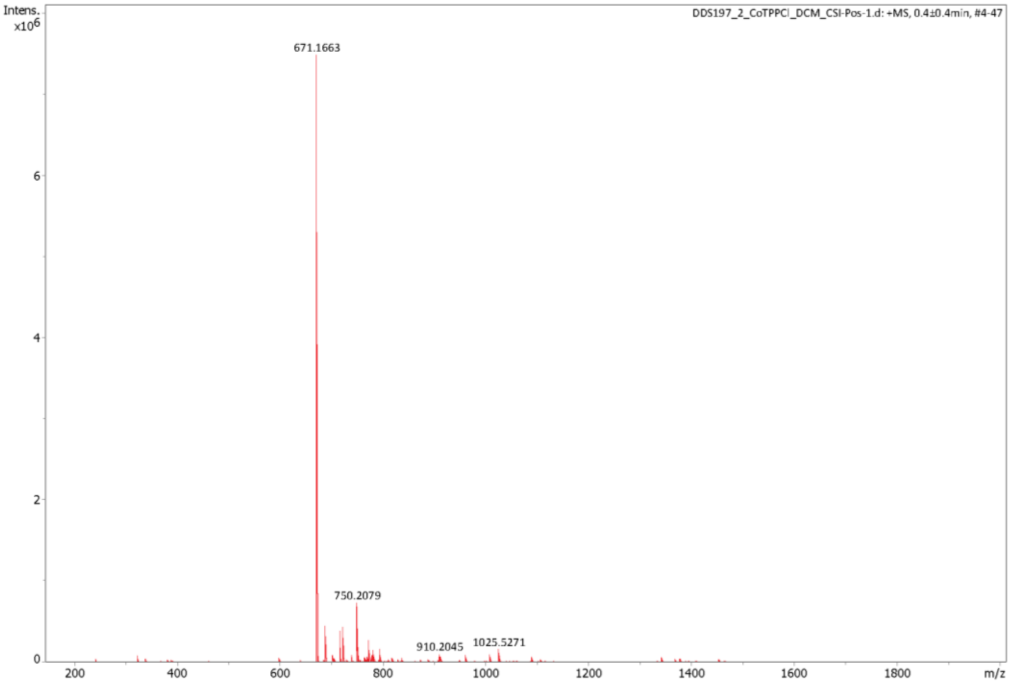


Figure S14. HRMS-CSI+ spectrum of [Co$^{III}$(TPP)Cl] at t = 126 days.

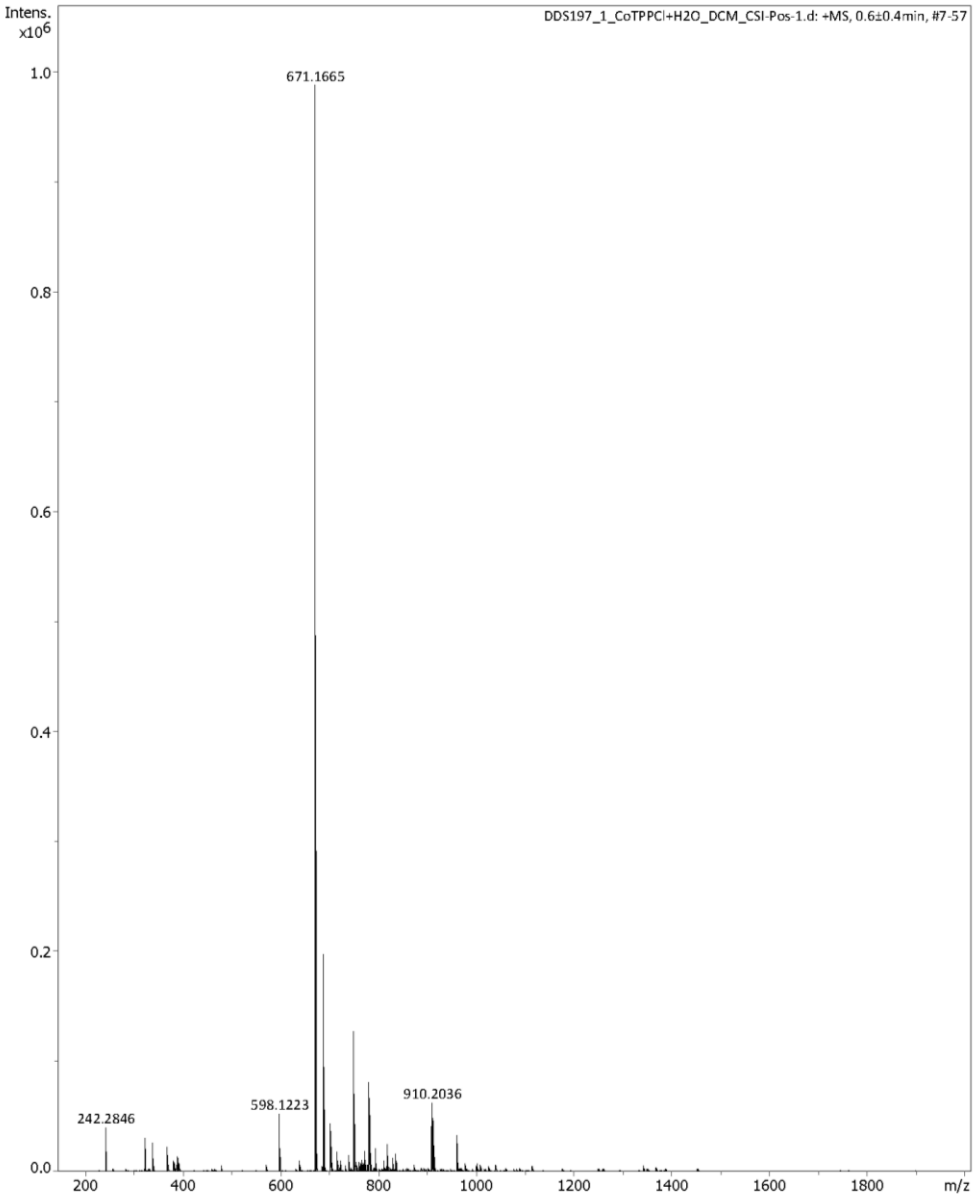


Figure S15. HRMS-CSI+ spectrum of [Co$^{III}$(TPP)Cl] at t = 126 days + 10 µL $H_2O$.

## 4. EPR studies

### General procedure EPR studies

To a flame-dried 5 mL Schlenk flask, [$Co^{III}$(TPP)Cl] (0.005 mmol, 0.1 eq.), KO*t*Bu (0.11 mmol, 2.2 eq.) and dimethyl malonate (0.05 mmol, 1 eq.) were mixed in toluene-$d_8$. The dark red mixture was freeze-pump-thawed, filtered, and transferred to a quartz EPR tube with benzophenone (0.03 mmol, 0.6 eq.) under an Argon flow. The EPR sample was irradiated with 370 nm (43 W) and was frozen immediately in liquid nitrogen, unless mentioned otherwise.

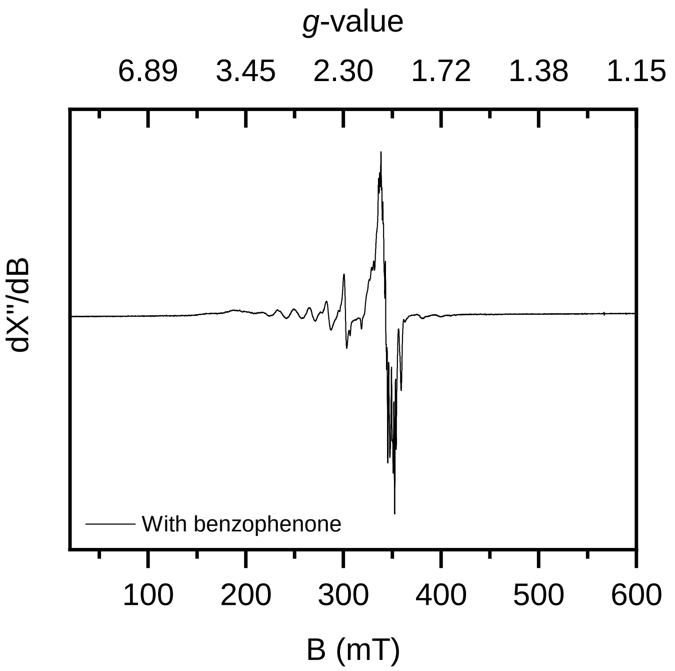


Figure S16. X-Band EPR spectrum obtained upon irradiation (370 nm) of *in situ* formed complex **1** in toluene-$d_8$ mixed with benzophenone at 40 K. Conditions: [$Co^{III}$(TPP)Cl] (3.1 mM), KO*t*Bu (68.5 mM, 22 eq.) and dimethyl malonate (31.2 mM, 10 eq.) were mixed in toluene-$d_8$. The suspension was stirred, sparged with Argon, and filtered before adding to benzophenone (74 mM, 24 eq.) inside a quartz EPR tube. Settings: 9.644576 MHz, 6.325 mW, modulation amplitude: 4 G.

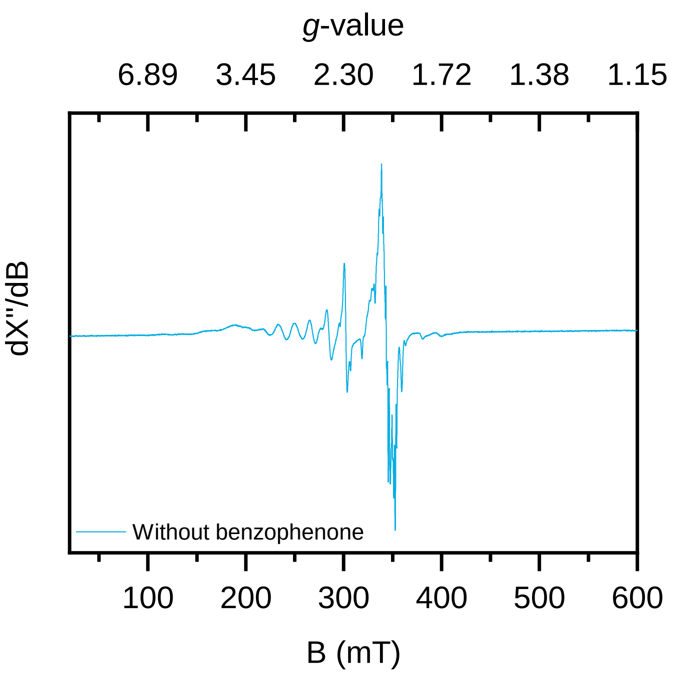


Figure S17. X-Band EPR spectrum obtained upon irradiation (370 nm) of *in situ* formed complex **1** in toluene-$d_8$ at 40 K. Conditions: [Co$^{III}$(TPP)Cl] (3.1 mM), KO*t*Bu (68.5 mM, 22 eq.) and dimethyl malonate (31.2 mM, 10 eq.) were mixed in toluene-$d_8$. The suspension was stirred, sparged with Argon, and filtered before adding to a quartz EPR tube. Settings: 9.645972 MHz, 6.325 mW, modulation amplitude: 4 G.

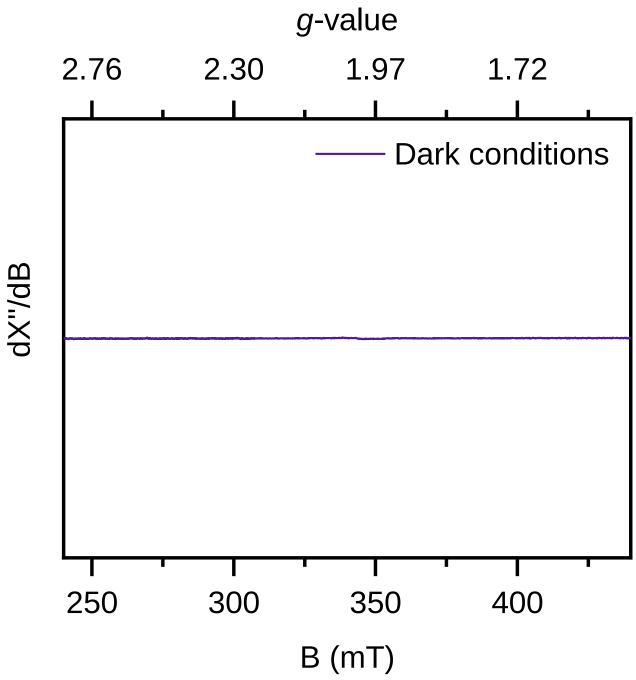


Figure S18. X-Band EPR spectrum of *in situ* formed complex **1** in $C_6H_6$ under dark conditions at 20 K. Conditions: [Co$^{III}$(TPP)Cl] (5.2 mM), KO*t*Bu (98.6 mM, 20 eq.) and dimethyl malonate (50.0 mM, 10 eq.) were mixed in $C_6H_6$. The suspension was stirred, sparged with Argon, and filtered before measuring the EPR sample. Settings: 9.644983 MHz, 0.6325 mW, modulation amplitude: 4 G.

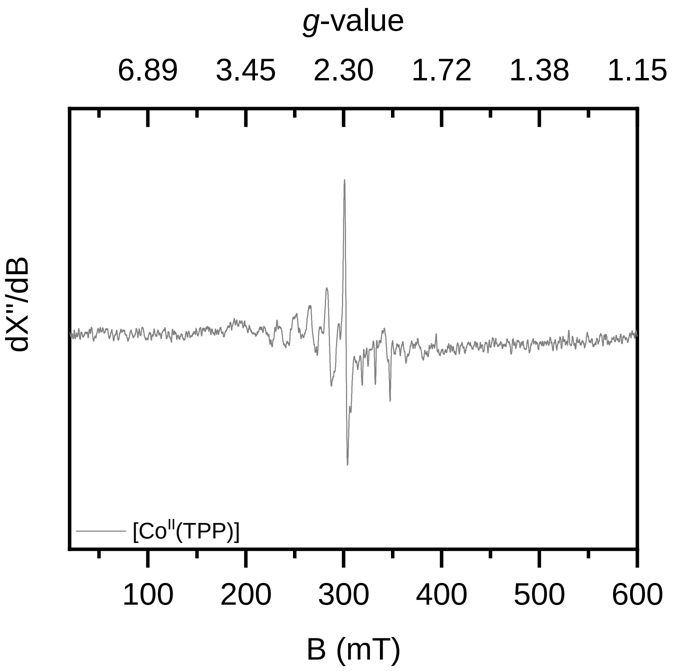


Figure S19. X-Band EPR spectrum of [Co$^{II}$(TPP)] in toluene-*d*$_8$ at 40 K. Conditions: [Co$^{II}$(TPP)] (4.9 mM), was dissolved in toluene-*d*$_8$. The solution was stirred, sparged with Argon, and filtered before adding to a quartz EPR tube. Settings: 9.647724 MHz, 20 mW, modulation amplitude: 6 G.

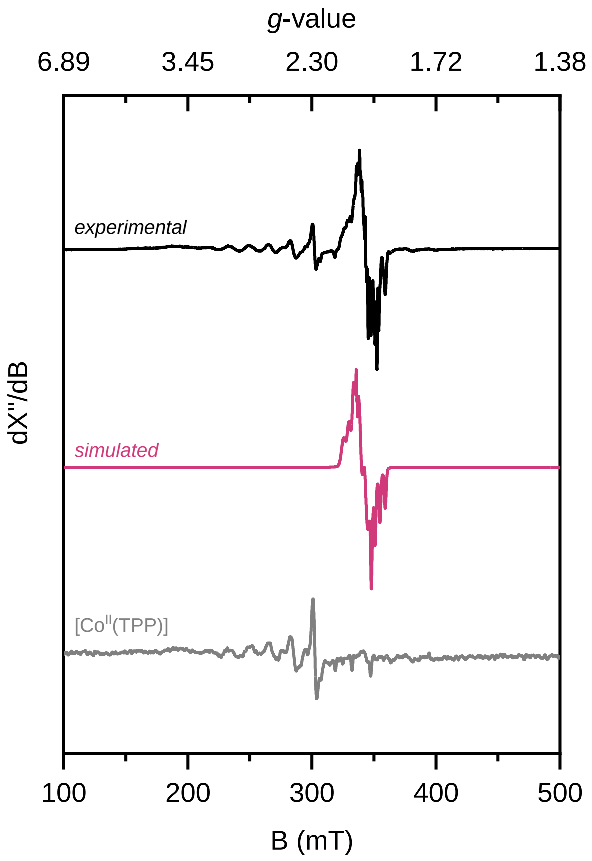


Figure S20. Comparison between the experimental EPR spectra of complex **1** after irradiation (black line) and [Co$^{II}$(TPP)] (grey line), and the simulated EPR spectrum of the cobalt(III) carbene radical complex (pink line).

**Table S5. Experimental EPR parameters of cobalt(III) carbene radical complex from spectral simulations.[a]**

| | |
|---|---|
| | |
| $g_{11}$ | 2.0177 |
| $g_{22}$ | 1.9880 |
| $g_{33}$ | 2.0465 |
| $g_{av}$ | 2.0174 |
| | |
| $A^{59Co}_{11}$ | 10 |
| $A^{59Co}_{22}$ | 22 |
| $A^{59Co}_{33}$ | 130 |
| $A^{59Co}_{av}$ | 54 |
| | |
| *Euler angles* | -31, 39, 73 |
| | |
| *S* | 0.5 |
| *lw* | 0.31, 0.41 |
| *Hstrain* | 50, 0, 0 |
| *gStrain* | 0.014, 0.005, 0 |
| *Astrain* | 25, 5, 25 |

[a]Spectral simulations performed with Easyspin,[15] using the cwEPR plugin.[16] Hyperfine couplings in MHz. Parameters based on previously reported calculations and used for the spectral simulations.[17]

# 5. NMR studies

## General procedure NMR studies

To a flame-dried 10 mL Schlenk flask, complex **1** (Figure S21, 0.00137 mmol, 1 eq.), benzophenone (Figure S22, 0.002 mmol, 1.5 eq.) and benzene (2 µL) as internal standard were dissolved in $CD_2Cl_2$ (0.5 mL). The dark red solution was freeze-pump-thawed and transferred to a *J*-young NMR tube after filtration. The tube was kept under dark conditions until the NMR measurements were performed. All NMR samples were irradiated with a 390-500 nm light source via a light probe attached to the bottom of the NMR machine. To determine the concentration of complex **1**, signals at -3.16 (1H) and 1.82 (6H) ppm were integrated. To determine the concentration of [$Co^{II}$(TPP)], signals at 9.62 (12H) and 12.85 (8H) ppm were integrated.

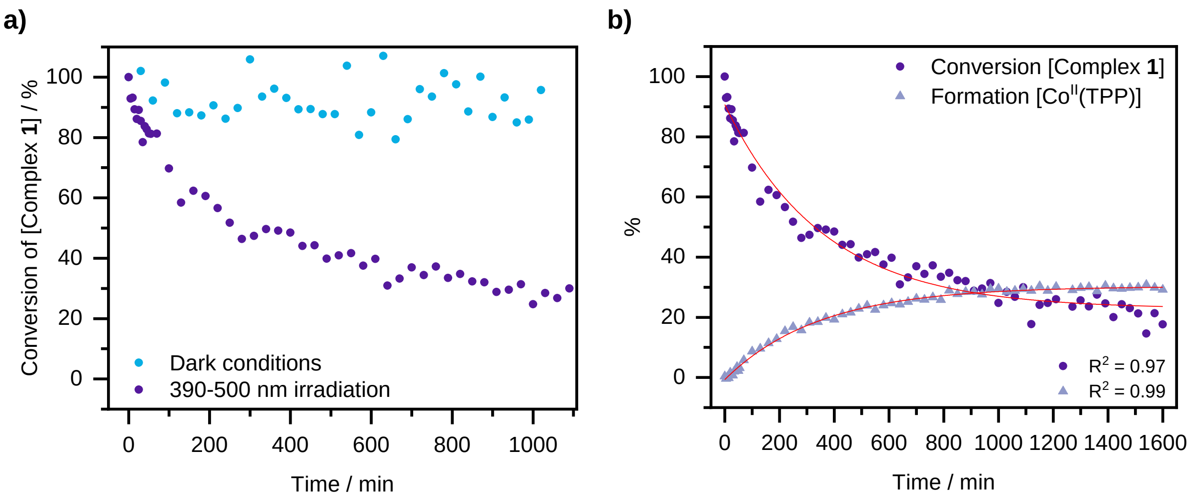


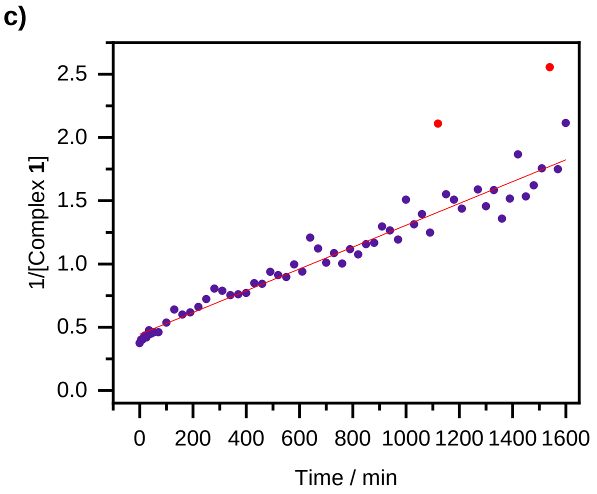


| | |
|---|---|
| Equation | y = a + b*x |
| Plot | 2nd order |
| Weight | No Weighting |
| Intercept | 0.44398 ± 0.01902 |
| Slope | 8.62326E-4 ± 2.30224 |
| Residual Sum of Squar | 0.48826 |
| Pearson's r | 0.97961 |
| R-Square (COD) | 0.95964 |
| Adj. R-Square | 0.95896 |

Figure S21. a) Conversion of complex **1** under dark conditions (blue, 2.75 mM) and upon 390-500 nm light irradiation (purple, 2.68 mM) in DCM-$d_2$. b) Conversion of complex **1** and formation of [$Co^{II}$(TPP)] upon 390-500 nm light irradiation in DCM-$d_2$. c) 1/[Complex **1**] plotted against time. Outliers in red do not contribute to the fitting.

Figure S22 shows the conversion of complex **1** in presence of benzophenone over time. Initially, an increase in rate was expected when benzophenone was present in the NMR sample. However, the sample was irradiated with a 390-500 nm light source, a region in which benzophenone does not absorb. The effect of benzophenone on the rate of the conversion of complex **1** can therefore not be concluded based on this NMR experiment. We do observe an increase in yield when benzophenone is present (Table S1, entry 1 and entry 4), proving that benzophenone has a positive effect on the formation of cyclopropane in the cyclopropanation reaction.

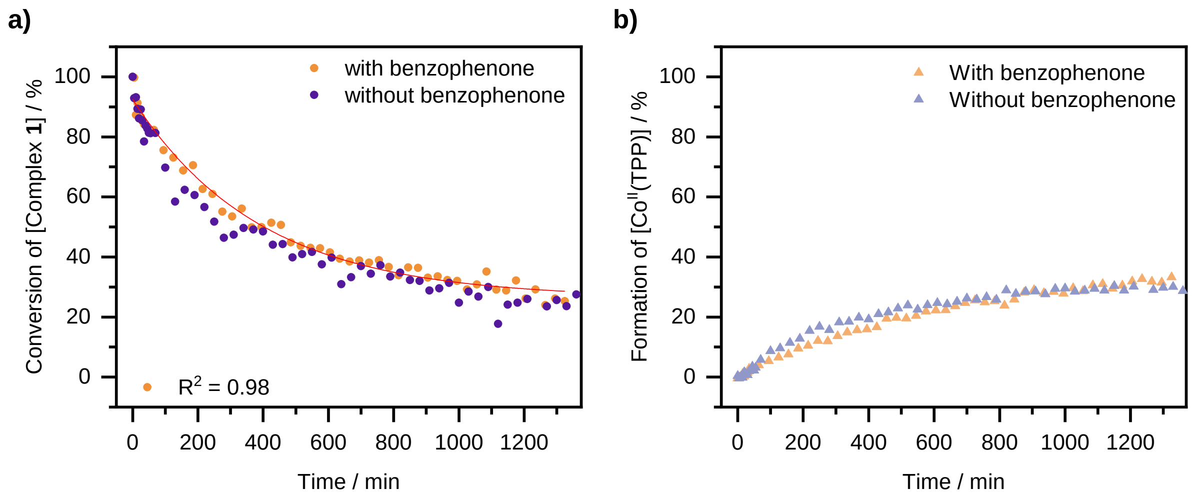


Figure S22. a) Conversion of complex **1** and b) formation of [Co$^{II}$(TPP)] with benzophenone (orange) and without benzophenone (purple) upon 390-500 nm light irradiation. Conditions with benzophenone: complex **1** (2.87 mM) and benzophenone (3.74 mM) in DCM-$d_2$.

## 6. UV-Vis studies

### General procedure UV-Vis studies

The UV-Vis spectrum of complex **1** was also measured over time (Figure S23). Complex **1** (0.690 mg) was dissolved in DCM (10 mL) inside a $N_2$-filled glovebox. The red solution was diluted to obtain a concentration of 21.5 µM and it was transferred to a *J*-young UV-Vis cuvette. The UV-Vis cuvette was kept in the dark before the measurements started. In between the measurements, the cuvette was irradiated with a green light source (525 nm, 43 W) for a defined period of time.

Under dark conditions, the UV-Vis spectrum of complex **1** remains constant (Figure S23a). Next, we irradiated the sample with 525 nm (43 W) (Figure S23b), because this wavelength is the lowest energy at which complex **1** still absorbs light. After 5 minutes, the shoulder at 375 nm disappeared and a blue shift of the Soret-band (initially 413 nm) was observed with a higher intensity. After 2.5 hours, the Soret-band lowered in intensity again and showed a stronger blue shift to 407 nm. The Q-band moved from 525 nm to 545 nm. Complex **1** clearly converts to other species upon irradiation of green light. While the nature of these species cannot be derived from the UV-Vis spectra, it is most likely that a mixture of species is formed, including [$Co^{II}$(TPP)] and the cobalt(III) carbene radical complex.

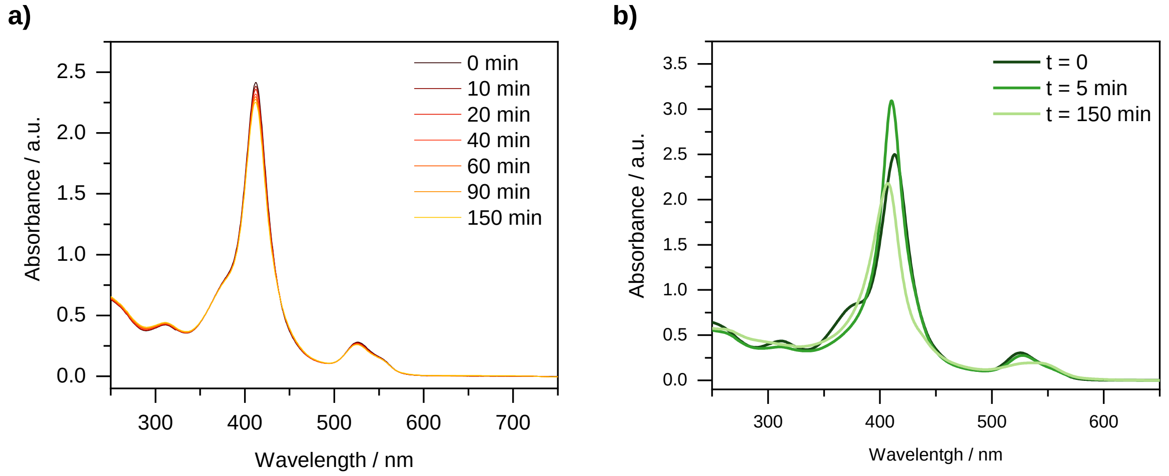


Figure S23. a) UV-Vis spectrum of complex **1** (21.5 µM) in DCM under dark conditions. b) UV-Vis spectrum of complex **1** (21.5 µM) in DCM upon 525 nm (43 W) light irradiation measured over time. All spectra were normalized at 725 nm.

## 7. Computational details

**Table S6.** Graphical representation of all optimized structures and Mulliken spin density plots of all optimized structures. Isovalue = 0.004, $\alpha$ = pink, $\beta$ = green.

| | **Structure** | **Mulliken spin density plots** |
|---|---|---|
| **Dimethyl malonate** | 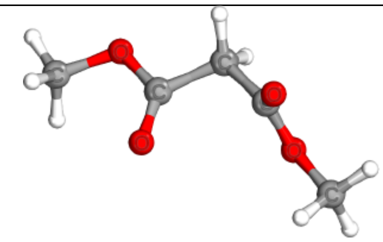 | – |
| **Dimethyl malonate radical** | 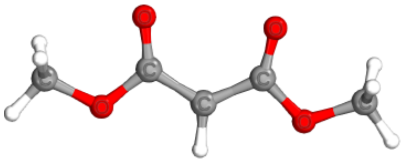 | 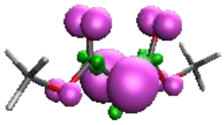 |
| **[$Co^{II}$(TPP)]** | 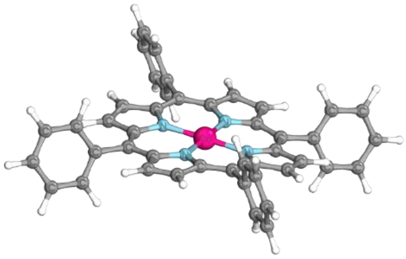 | 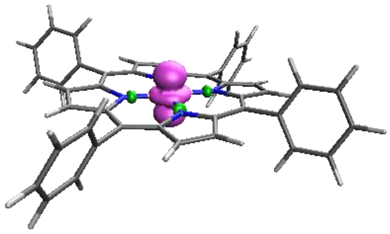 |
| **Complex 1: [$Co^{III}$(TPP)(CH($CO_2$Me)$_2$]** | 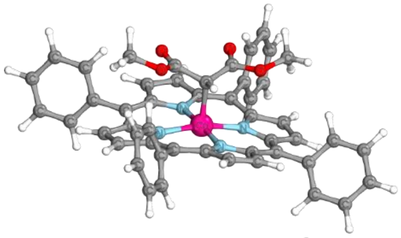 | – |
| **Cobalt(III) Carbene Radical Complex 2: [$Co^{III}$(TPP)(C($CO_2$Me)$_2$]** | 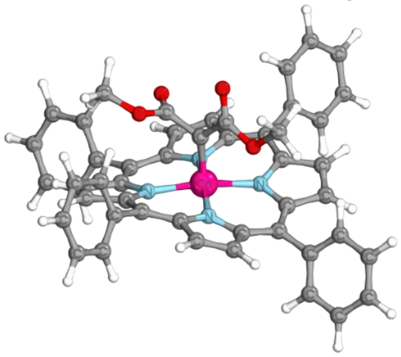 | 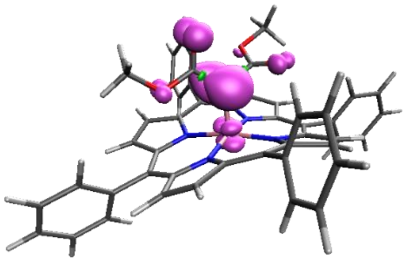 |

**Table S7.** Calculated $<s^2>$, negative frequencies and energies (Hartree) of all optimized structures at 298 K.

| Molecule | Negative frequencies ($cm^{-1}$) | $<s^2>$ | SCF | ZPE correction | Enthalpy correction | Entropy correction | Gibbs free energy |
|---|---|---|---|---|---|---|---|
| Dimethyl malonate | None | – | -496.50974 | 0.12872 | 0.14008 | 0.09104 | -496.4187 |
| Dimethyl malonate radical | None | 0.7550 | -495.85085 | 0.11554 | 0.12684 | 0.07762 | -495.77323 |
| $[Co^{II}(TPP)]$ | None | 0.7616 | -3296.5794 | 0.58051 | 0.61927 | 0.50709 | -3296.07235 |
| Complex **1** $[Co^{III}(TPP)(CH(CO_2Me)_2]$ | None | – | -3792.51384 | 0.7005 | 0.7499 | 0.61609 | -3791.89775 |
| Cobalt(III) carbene radical $[Co^{III}(TPP)(C(CO_2Me)_2]$ | None | 0.7555 | -3791.8568 | 0.68764 | 0.73733 | 0.60194 | -3791.25488 |

## 8. References


1. Fulmer, G. R.; Miller, A. J. M.; Sherden, N. H.; Gottlieb, H. E.; Nudelman, A.; Stoltz, B. M.; Bercaw, J. E.; Goldberg, K. I. NMR Chemical Shifts of Trace Impurities: Common Laboratory Solvents, Organics, and Gases in Deuterated Solvents Relevant to the Organometallic Chemist. *Organometallics* **2010**, *29*, 2176–2179. DOI: 10.1021/om100106e
2. TURBOMOLE Version 7.4.1. TURBOMOLE Gmbh: Karlsruhe, Germany 2019.
3. PQS Version 2.4; Parallel Quantum Solutions: Fayetteville, AR, USA, **2001**.
4. Baker, J. An Algorithm for the Location of Transition States. *J. Comput. Chem.* **1986**, *7*, 385–395. DOI: 10.1002/jcc.540070402
5. Budzelaar, P. H. M. Geometry Optimization Using Generalized, Chemically Meaningful Constraints. *J. Comput. Chem.* **2007**, *328*, 2226–2236. DOI: 10.1002/jcc.20740
6. Grimme, S. Density Functional Theory with London Dispersion Corrections. *Wiley Interdiscip. Rev. Comput. Mol. Sci.* **2011**, *1*, 211–228. DOI: 10.1002/wcms.30
7. (a) Available at www.iboview.org. (b) Knizia, G.; Klein, J. E. M. N. Electron Flow in Reaction Mechanisms - Revealed from First Principles. *Angew. Chemie Int. Ed.* **2015**, *54*, 5518–5522. DOI: 10.1002/anie.201410637 (c) Knizia, G. Intrinsic Atomic Orbitals: An Unbiased Bridge between Quantum Theory and Chemical Concepts. *J. Chem. Theory Comput.* **2013**, *9*, 4834–4843. DOI: 10.1021/ct400687b
8. Available free of charge at http://iqmol.org/.
9. (a) Becke, A. D. Density-Functional Exchange-Energy Approximation with Correct Asymptotic Behavior. *Phys. Rev. A* **1988**, *38*, 3098–3100. DOI: 10.1063/1.1749835 (b) Perdew, J. P. Density-Functional Approximation for the Correlation Energy Of the Inhomogeneous Electron Gas. *Phys. Rev. B* **1986**, *33*, 8822–8824. DOI: 10.1103/physrevb.33.8822 (c) Perdew, J. P. Erratum: Density-Functional Approximation for the Correlation Energy of the Inhomogeneous Electron Gas. *Phys. Rev. B* **1986**, *34*, 7406–7406. DOI: 10.1103/physrevb.34.7406
10. (a) Weigend, F.; Häser, M.; Patzelt, H.; Ahlrichs, R. RI-MP2: Optimized Auxiliary Basis Sets and Demonstration of Efficiency. *Chem. Phys. Lett.* **1998**, *294*, 143–152. DOI: 10.1016/s0009-2614(98)00862-8 (b) Weigend, F.; Ahlrichs, R. Balanced Basis Sets of Split Valence, Triple Zeta Valence and Quadruple Zeta Valence Quality for H to Rn: Design and Assessment of Accuracy. *Phys. Chem. Chem. Phys.* **2005**, *7*, 3297–3305. DOI: 10.1039/b508541a
11. Scheibel, M. G.; Abbenseth, J.; Kinauer, M.; Heinemann, F. W.; Würtele, C.; de Bruin, B.; Schneider, S. Homolytic N−H Activation of Ammonia: Hydrogen Transfer of Parent Iridium Ammine, Amide, Imide, and Nitride Species. *Inorg. Chem.* **2015**, *54*, 9290 – 9302. DOI: doi/10.1021/acs.inorgchem.5b00829
12. Gao, Y.; DeYonker, N. J.; Chauncey Garrett III, E.; Wilson, A. K.; Cundari, T. R.; Marshall, R. *J. Phys. Chem. A.* **2009**, *113*, 6955–6963. DOI: doi/10.1021/jp901314y
13. Agapio, F.; Nunes, P. M.; Costa Cabral, B. J.; Borges dos Santos, R. M.; Martinho Simõnes, J. A. *J. Org. Chem.* **2007**, *72*, 8770–8779. DOI: 10.1021/jo701397r
14. Sakurai, T.; Yamamoto, K.; Naito, H.; Nakamoto, N. The Crystal and Molecular Structure of Chloro-abgd-tetraphenylporphinacobalt(III). *Bull. Chem. Soc. Jpn.* **1976**, *49*, 3042–3046. DOI: doi.org/10.1246/bcsj.49.3042
15. Stoll, S; Schweiger, A. EasySpin, a Comprehensive Software Package for Spectral Simulation and Analysis in EPR. *J. Magn. Reson.* **2006**, *178*, 42–55. DOI: 10.1016/j.jmr.2005.08.013
16. Thomas Casey. cwEPR (https://www.mathworks.com/matlabcentral/fileexchange/73292–cwepr), MATLAB Central File Exchange (**2022**).
17. Epping, R. F. J.; Hoeksma, M. M.; Bobylev, E. O.; Mathew, S.; de Bruin, B. Cobalt(II)–tetraphenylporphyrin-catalysed carbene transfer from acceptor–acceptor iodonium ylides via N-enolate–carbene radicals. *Nat. Chem.* **2022**, *14*, 550–557. DOI: 10.1038/s41557-022-00905-4
